# Target-Based Discovery of an Inhibitor of the Regulatory Phosphatase PPP1R15B

**DOI:** 10.1016/j.cell.2018.06.030

**Published:** 2018-08-23

**Authors:** Agnieszka Krzyzosiak, Anna Sigurdardottir, Laura Luh, Marta Carrara, Indrajit Das, Kim Schneider, Anne Bertolotti

**Affiliations:** 1 Neurobiology Division, MRC Laboratory of Molecular Biology, Francis Crick Avenue, Cambridge CB2 0QH, UK

**Keywords:** protein phosphatase 1, protein quality control, proteostasis, protein misfolding, eukaryotic initiation factor-2, PPP1R15B, neurodegenerative diseases, stress response, Huntington’s disease, drug discovery

## Abstract

Protein phosphorylation is a prevalent and ubiquitous mechanism of regulation. Kinases are popular drug targets, but identifying selective phosphatase inhibitors has been challenging. Here, we used surface plasmon resonance to design a method to enable target-based discovery of selective serine/threonine phosphatase inhibitors. The method targeted a regulatory subunit of protein phosphatase 1, PPP1R15B (R15B), a negative regulator of proteostasis. This yielded Raphin1, a selective inhibitor of R15B. In cells, Raphin1 caused a rapid and transient accumulation of its phosphorylated substrate, resulting in a transient attenuation of protein synthesis. *In vitro*, Raphin1 inhibits the recombinant R15B-PP1c holoenzyme, but not the closely related R15A-PP1c, by interfering with substrate recruitment. Raphin1 was orally bioavailable, crossed the blood-brain barrier, and demonstrated efficacy in a mouse model of Huntington’s disease. This identifies R15B as a druggable target and provides a platform for target-based discovery of inhibitors of serine/threonine phosphatases.

## Highlights

• A method to enable target-based discovery of selective phosphatase inhibitors
• Raphin1 inhibits PPP1R15B, transiently lowers translation, and improves proteostasis
• Raphin1 is orally available and crosses the blood-brain barrier
• Raphin1 decreases deficits in a mouse model of Huntington’s disease

## Graphical Abstract

**Figure:**
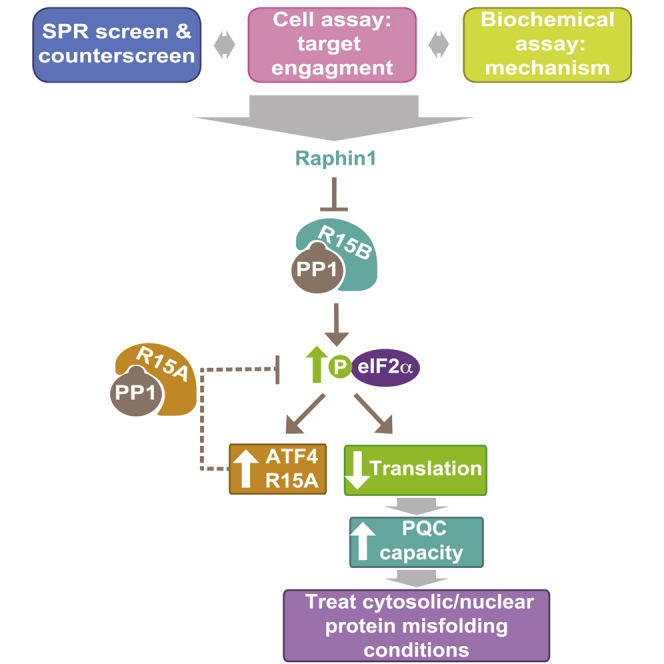

## Teaser

An orally available selective phosphatase inhibitor improves proteostasis and diminishes deficits in a mouse model of Huntington’s disease.

## Introduction

A vital response to stresses consists of phosphorylating the α subunit of eukaryotic translation initiation factor 2 (eIF2α) on Serine 51 to slow protein synthesis, thereby sparing cellular resources to cope with challenges (Ron and Harding, 2007). Under physiological conditions, phosphorylation of eIF2α constantly fluctuates to adapt protein synthesis rates to changing needs (Pilla et al., 2017). Extreme perturbations of this rheostatic regulation through a lack (Harding et al., 2001, Scheuner et al., 2001) or an excess (Srivastava et al., 1998) of eIF2α phosphorylation are deleterious. In mammals, two heterodimeric eIF2α holophosphatases maintain low levels of eIF2α phosphorylation: each comprises a regulatory subunit, the stress-inducible PPP1R15A (R15A), or the constitutive PPP1R15B (R15B), which is bound to the catalytic subunit protein phosphatase 1 (PP1c) (Jousse et al., 2003, Novoa et al., 2001). The R15A inhibitor Guanabenz (GBZ) (Tsaytler et al., 2011) and its derivative Sephin1 (Das et al., 2015) were discovered in phenotypic assays by virtue of their ability to protect cells from otherwise lethal protein misfolding stress in the endoplasmic reticulum (ER stress). GBZ and Sephin1 selectively inhibit the stress-induced R15A and thereby prolong eIF2α phosphorylation and the resultant transient attenuation of protein synthesis induced by endoplasmic reticulum (ER) stress (Das et al., 2015, Tsaytler et al., 2011). Because a large fraction of the proteostasis system is normally committed to newly synthesized proteins, the cellular capacity to handle misfolded proteins increases when the biosynthetic load decreases (Pilla et al., 2017). Thus, by prolonging eIF2α phosphorylation and translation attenuation, R15A inhibition in turn increases chaperone capacity to misfolded proteins, thereby enhancing proteostasis (Tsaytler et al., 2011). This restores fitness of ER-stressed cells in culture (Tsaytler et al., 2011) and in mice (Das et al., 2015).

GBZ and Sephin1 have shown the benefit of R15A inhibition. However, because R15A’s expression is restricted to limited conditions, such as ER stress, and because GBZ and Sephin1 are selective inhibitors of R15A, it is anticipated that the use of R15A inhibitors will be restricted to conditions in which R15A is induced. We reasoned that since R15B is functionally related to R15A, selective inhibition of R15B could circumvent the limitations associated with R15A inhibition, while conferring the same proteostatic benefit. Supporting this idea, knockdown of R15B protects cells from diverse stresses (Jousse et al., 2003). Many human diseases associated with accumulation of misfolded proteins could be in principle corrected by enhancing the capacity of the proteostasis network (Balch et al., 2008). In this context, R15B is an attractive potential therapeutic target. However, it is unknown whether it is possible to selectively inhibit R15B.

The bulk of protein phosphorylation events occurs on serine and threonine residues, and PP1c is the prevalent phosphatase accounting for the majority of serine/threonine dephosphorylation (Brautigan, 2013, Cohen, 2002, Heroes et al., 2013, Hubbard and Cohen, 1993, Roy and Cyert, 2009). PP1c is a single-domain protein that in cells is not free but is part of a dimeric (or a trimeric) holophosphatase complex with one subunit (or two subunits) among an array of very diverse regulatory subunits (Virshup and Shenolikar, 2009). As a consequence, catalytic inhibitors of PP1c are not selective because they inhibit hundreds of holophosphatases and as a result, PP1c inhibitors are toxic to cells (De Munter et al., 2013, Tsaytler and Bertolotti, 2013). Therefore, drug discovery efforts have traditionally neglected phosphatases.

The discovery of Guanabenz and Sephin1 through a phenotypic screen has revealed that R15A, a regulatory subunit of PP1c, can be selectively inhibited (Das et al., 2015, Tsaytler and Bertolotti, 2013, Tsaytler et al., 2011). While in theory, other regulatory phosphatases could be inhibited in the same way, it remained unknown whether this was feasible or not. Moreover, there were no methods available to enable the identification of selective inhibitors of regulatory subunits, a challenge we set out to overcome. Finding a way to selectively inhibit phosphatases is important because there are about 200 phosphatases that control all aspects of cell biology and that could be in principle exploited as drug targets. Here, we present a platform of assays that enabled the target-based discovery of an inhibitor of R15B.

## Results

### A Surface-Plasmon-Resonance-Based Method with Reconstituted Holophosphatases

**Figure S1:**
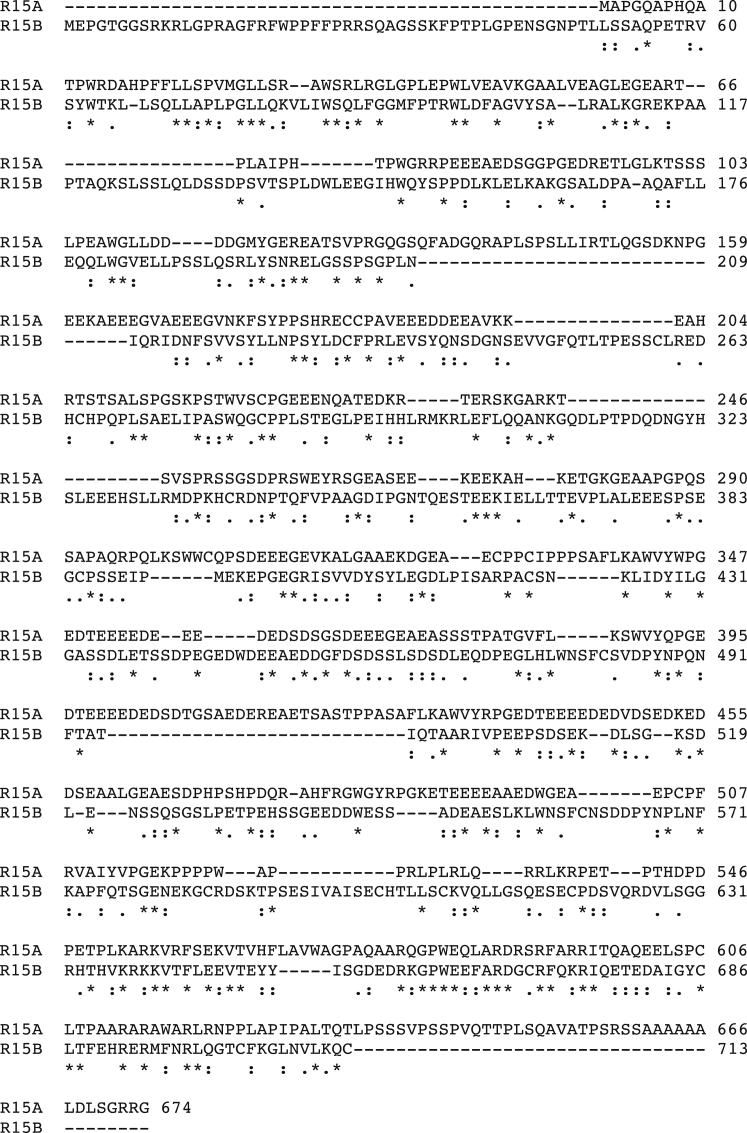
Sequence Alignment of Human R15A and R15B, Related to Figure 1 Sequences of human R15A and R15B using CLUSTAL O (version 1.2.1) shows 23.8% sequence identity (using a Percent Identity Matrix) (Sievers et al., 2011).

**Figure 1:**
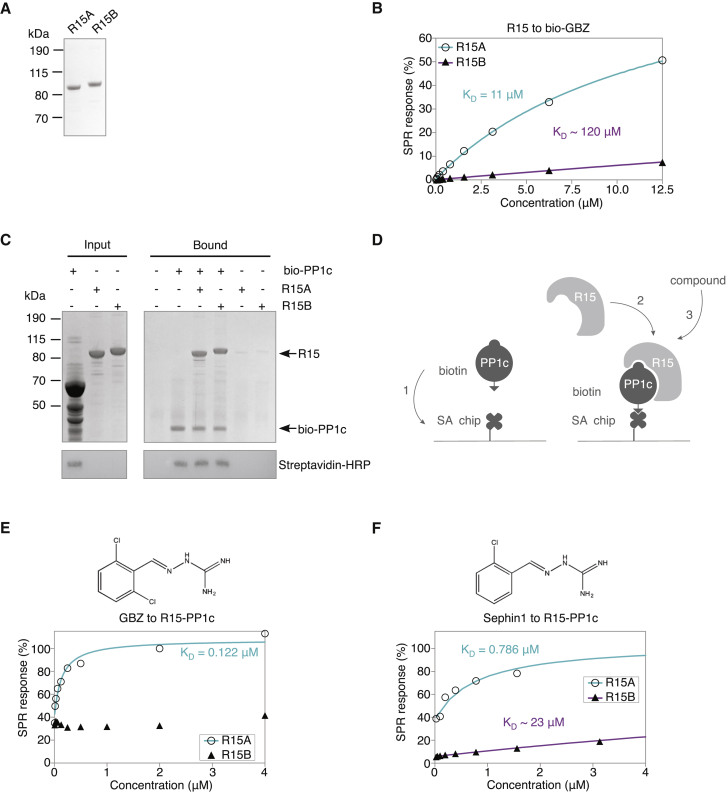
An SPR-Based Assay with Reconstituted R15 Holophosphatases Measures Affinities of R15A Inhibitors (A) Coomassie-stained gel showing recombinant proteins used in (B): MBP-R15A^325-636^-His and MBP-R15B^340-698^-His. (B) Normalized steady-state binding curves from SPR showing binding of R15A (○ cyan) and R15B (▲ magenta) to bio-GBZ immobilized on the streptavidin sensor chip surface. Bio-GBZ (biotinylated GBZ) is an R15A inhibitor as potent as GBZ (Tsaytler et al., 2011). (C) Coomassie-stained gel showing recombinant biotinylated PP1c (bio-PP1c; partially purified) and purified recombinant R15s (Input). Bio-PP1c, captured on neutravidin beads, bound R15A and R15B (Bound). Lower panel: immunoblot showing bio-PP1c. (D) Cartoon depicting the reconstitution of R15 holophosphatases (R15A^325-636^-PP1c and R15B^340-698^-PP1c; see STAR Methods) on a streptavidin (SA) SPR chip. (E and F) Normalized SPR steady-state binding curves showing binding of GBZ (E) or Sephin1 (F) to R15A-PP1c (○ cyan) and R15B-PP1c (▲ magenta) reconstituted on the streptavidin sensor chip surface. Representative results of three independent experiments are shown. See also Figures S1 and S2.

**Figure S2:**
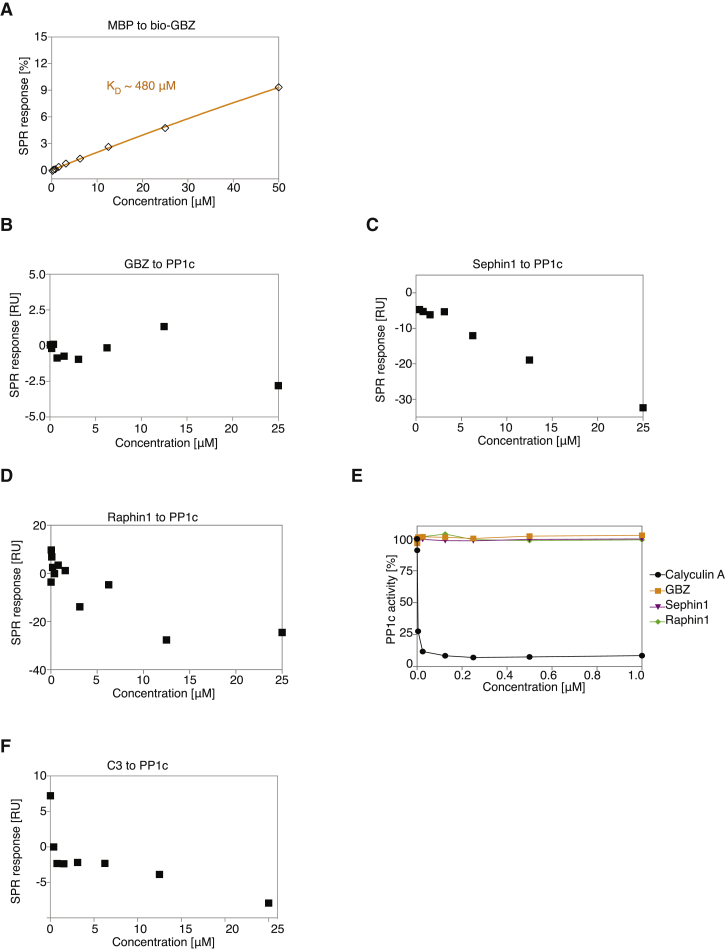
Selectivity of Compounds, Related to Figures 1 and 2 (A) Normalized steady-state binding curves from SPR showing binding of MBP (maltose binding protein, a control) (◇ orange) to bio-GBZ immobilized on the streptavidin sensor chip surface. K_D_ values were calculated with a steady-state affinity model by the Biacore T200 analysis software (Biaevaluation Version 1.0) by plotting equilibrium response units against protein concentration. For comparing the results the steady-state binding curves were normalized against R_max_ (maximum binding capacity of the surface based on the respective steady-state curve). (B, C, D, and F) Response units plotted against compound concentration showed no binding of GBZ (B), Sephin1 (C), Raphin1 (D) or compound C3 (F) to PP1c (■) immobilized on the streptavidin sensor chip surface. (E) Raphin1 does not inhibit PP1c. Dephosphorylation of difluoro-4-methylumbelliferyl phosphate by PP1c is inhibited by Calyculin A but not by GBZ, Sephin1 or Raphin1. Representative results of three independent experiments are shown in each panel.

Recently, we developed assays that enabled the functional characterization of the eIF2α phosphatases and revealed that GBZ and Sephin1 induce a selective conformational change in R15A, thereby inhibiting its function (Carrara et al., 2017). These assays are valuable for functional studies but currently lack the sensitivity and dynamic range required for small molecule screening and ranking. To overcome this limitation, we turned to surface plasmon resonance (SPR) and first aimed to measure the binding affinities of R15A inhibitors to their target, in comparison to R15B. Note that although R15A and R15B are functionally related, they only share 23.8% sequence identity (Figure S1), exemplifying the known diversity of PP1c regulatory subunits (Heroes et al., 2013, Virshup and Shenolikar, 2009). We found that a functional recombinant R15A^325–636^ fragment (Carrara et al., 2017) bound to a biotinylated derivative of GBZ immobilized on a SPR chip with a much higher affinity than a functional recombinant R15B^340–698^ fragment (Carrara et al., 2017) (Figures 1A, 1B, and S2A), confirming the selectivity of GBZ for R15A (Tsaytler et al., 2011). However, the measured 11 ± 0.6 μM affinity of R15A for biotinylated GBZ (Figure 1B) was incompatible with the submicromolar potency of GBZ in cells (Tsaytler et al., 2011). Assays relying on isolated regulatory subunits may have some limitations, because these proteins are intrinsically disordered and are believed to fold upon binding to PP1c (Boens et al., 2013). Therefore, we next reconstituted R15-PP1c holophosphatases on a SPR chip. We modified the functional and selective eIF2α holophosphatases R15A^325–636^-PP1c and R15B^340–698^-PP1c previously described (Carrara et al., 2017) using biotinylated PP1c (see STAR Methods) to facilitate purification and capture. The R15-PP1c complexes were purified by affinity purification on a neutravidin resin (Figure 1C). The R15-PP1c holophosphatases were next reconstituted on a SPR streptavidin sensor chip in two steps and used to test binding to known inhibitors (Figure 1D). GBZ and Sephin1 strongly bound to R15A-PP1c, but did not bind or only weakly bound to R15B-PP1c (Figures 1E and 1F) and did not measurably bind to PP1c alone (Figures S2B and S2C), confirming their selectivity for R15A. The measured steady-state affinities of GBZ and Sephin1 for R15A-PP1c were 0.122 ± 0.009 μM and 0.786 ± 0.036 μM, respectively (Figures 1E and 1F), compatible with their submicromolar potency of the inhibitors in cell-based assays (Das et al., 2015, Tsaytler et al., 2011) and *in vivo*. Thus, SPR experiments conducted with holophosphatases measured relevant affinities of R15A inhibitors.

### Small-Molecule Screening with the Holophosphatase SPR Method Identifies Raphin1

**Figure 2:**
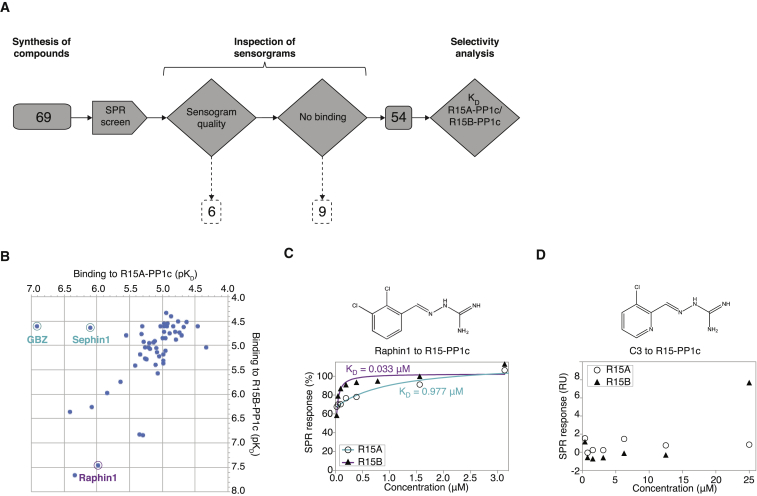
SPR-Based Screening with Reconstituted Holophosphatases Identifies Raphin1 (A) Overview of the screening strategy. Numbers in dark gray squares refer to the number of compounds screened and selected for the following step; numbers in dashed squares show the number of excluded compounds. The arrow represents the experimental assay, and diamonds represent analysis steps. During the data analysis, compounds were rejected based on sensorgram quality or no binding to either holophosphatase. (B) Results of the SPR screening showing binding affinities of 54 compounds to R15A-PP1c versus R15B-PP1c, plotted as pK_D_ values (negative logarithm of the dissociation constant). The 15 compounds that did not bind R15A-PP1c nor R15B-PP1c were not represented on the graph. To depict compounds only binding to R15A-PP1c on the graph, we arbitrarily set the dissociation constant for R15B-PP1c to 25 μM (the highest concentration used in the screen). (C) Normalized SPR steady-state binding curves showing binding of Raphin1 to R15A-PP1c (○ cyan) and R15B-PP1c (▲ magenta) reconstituted on the streptavidin sensor chip surface. (D) Response units plotted against compound concentration showed no binding of compound C3 to R15A-PP1c (○) or R15B-PP1c (▲) reconstituted on the streptavidin sensor chip surface. Representative results of three independent experiments are shown. See also Figure S2.

Having validated the method with known R15A inhibitors, we used it to search for molecules binding preferentially to R15B. Because Sephin1, a GBZ derivative, had a low binding affinity (>23 μM) to R15B, we wondered whether selective R15B inhibitors could be found in the GBZ chemical space. Therefore, we synthesized GBZ derivatives (to be reported elsewhere) and used the holophosphatase SPR method described above to search for molecules selectively binding to R15B. 69 compounds were screened (Figures 2A and 2B), yielding Raphin1 (rational inhibitor of a holophosphatase), an isomer of GBZ which bound strongly (K_D_ = 0.033 ± 0.02 μM) to the R15B-PP1c holophosphatase (Figures 2B and 2C). Raphin1 was ∼30-fold selective in binding R15B-PP1c over R15A-PP1c and did not bind to PP1c (Figure S2D). Thus, altering the position of the chloro-substitutions on GBZ remarkably switches its binding selectivity from R15A to R15B. In agreement with the binding studies, Raphin1 did not inhibit PP1c (Figure S2E), like GBZ and Sephin1 but unlike Calyculin A, a catalytic inhibitor of PP1c (McCluskey et al., 2002). Further validating the holophosphatase SPR method, compound C3, a GBZ derivative that was previously found inactive in cellular and biochemical assays (Carrara et al., 2017), had no detectable binding to R15-PP1c (Figure 2D) or PP1c (Figure S2F). Thus, SPR experiments conducted with two different recombinant holophosphatases provided a quantitative method to measure relevant steady-state binding affinities of the two known inhibitors of R15A and enabled the discovery of Raphin1, a new chemical entity preferentially binding to R15B.

### Raphin1 Inhibits R15B in Cells

**Figure S3:**
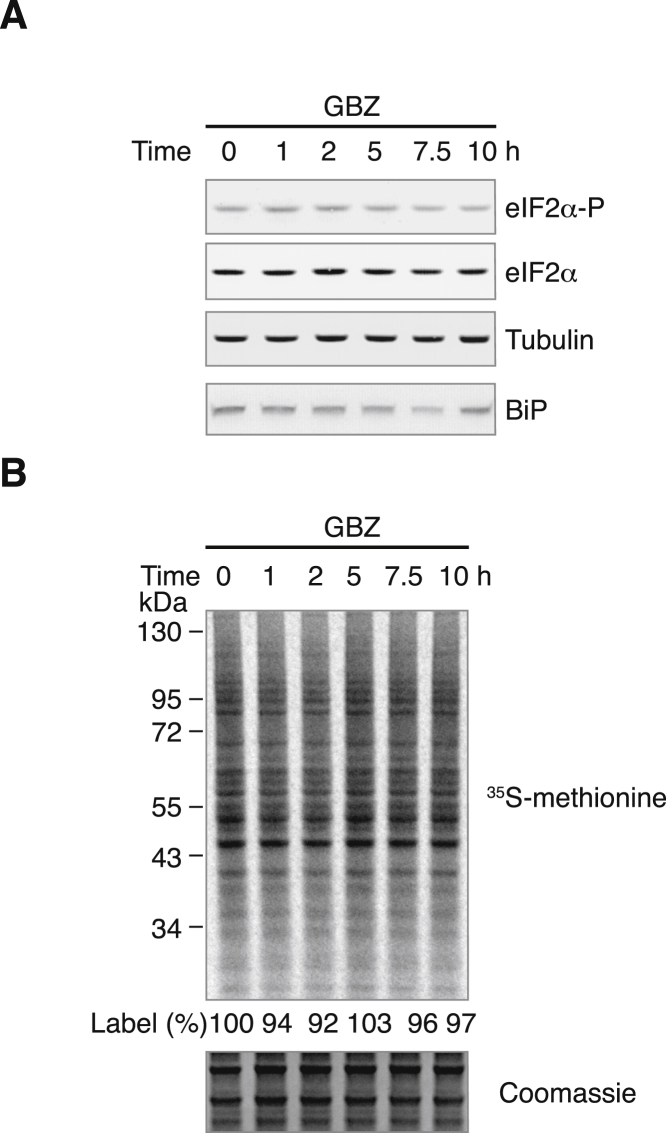
Guanabenz Has No Measurable Effects on eIF2α Phosphorylation and on the Rates of Protein Synthesis in Unstressed Cells, Related to Figure 3 (A) Immunoblots of the indicated proteins in HeLa cells lysates treated with GBZ at 10 μM for the indicated time. (B) Upper panel: Autoradiogram of newly synthesized proteins radiolabeled with ^35^S-methionine from HeLa cells lysates treated with GBZ at 10 μM for the indicated time. Lower panel: Coomassie staining of the gel. Representative results of three independent experiments are shown.

**Figure 3:**
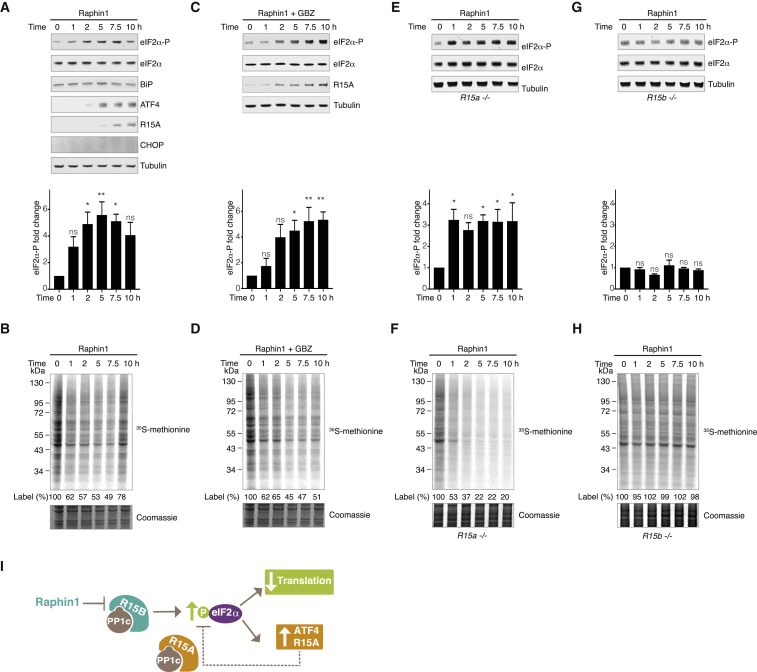
Raphin1 Inhibits R15B in Cells, Inducing a Transient Increase of eIF2α Phosphorylation and Attenuation of Protein Synthesis (A, C, E, and G) Top: immunoblots of the indicated proteins in HeLa (A and C), *R15a −/*− (E), or *R15b −/−* (G) cells lysates treated with the indicated compounds at 10 μM for the indicated time. Bottom: quantifications of eIF2α phosphorylation in immunoblots as shown above. Data are means ± SEM; n = 3. ^∗^p < 0.05; ^∗∗^p < 0.01 by an unpaired two-tailed Student’s t test in comparison to 0 hr time point. ns, not significant. (B, D, F, and H) Upper panel: autoradiogram of newly synthesized proteins radiolabeled with ^35^S-methionine in HeLa (B and D), *R15a −/−* (F), or *R15b −/−* (H) cells treated with the indicated compounds at 10 μM for the indicated time. Lower panel: Coomassie-stained gel. Representative results of three independent experiments are shown. (I) Cartoon illustrating the activity of Raphin1. See also Figures S3 and S4.

Having found that the measured steady-state affinities of GBZ and Sephin1 for R15A-PP1 were compatible with their potency and selectivity in cells, we then tested if Raphin1 could inhibit R15B in cells. We chose a concentration of 10 μM for Raphin1 because the dissociation constant measured by SPR (Figure 2C) predicted that this concentration should be sufficient to fully engage R15B in cells. As expected (Tsaytler et al., 2011), under basal conditions, GBZ had no measurable effects (Figure S3), because R15A is not induced in absence of stress (Novoa et al., 2001, Tsaytler et al., 2011). In contrast to GBZ, Raphin1 rapidly and transiently increased eIF2α phosphorylation and decreased protein synthesis (Figures 3A and 3B). Raphin1 did not increase the stress marker BiP (Figure 3A). This is expected for a R15B inhibitor because genetic inactivation of R15B does not induce BiP (Jousse et al., 2003). Transcripts encoding ATF4 and R15A are selectively translated when eIF2α is phosphorylated (Harding et al., 2000, Lee et al., 2009) and they were induced by Raphin1 (Figure 3A), unlike the pro-apoptotic protein CHOP (Figure 3A), which requires both ATF4 and another stress signaling pathway (Fusakio et al., 2016, Ma et al., 2002, Novoa et al., 2003).

**Figure S4:**
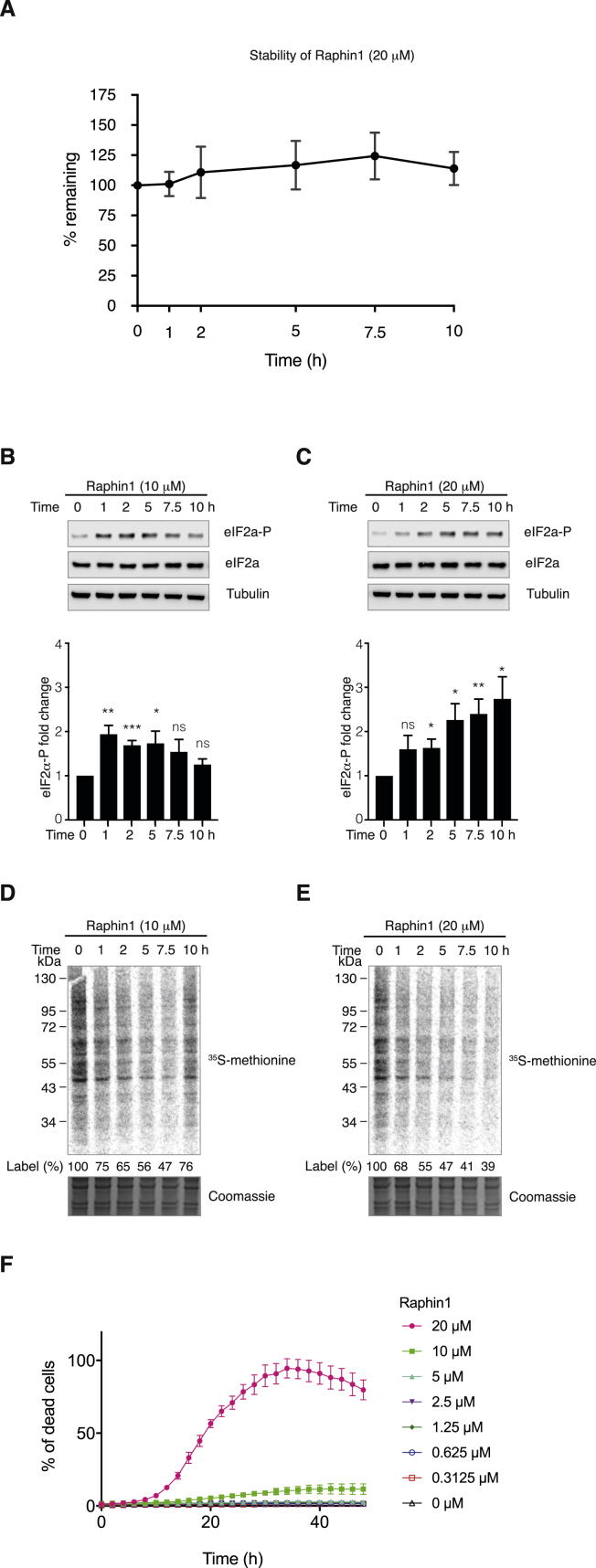
Effects of Raphin1 at 10 or 20 μM, Related to Figure 3 (A) Measurement of Raphin1 stability in cell culture media over time at 37°C. Data are means ± SEM, n = 2. (B and C) Immunoblots (top) of the indicated proteins in HeLa cells lysates treated with Raphin1 at 10 (B) or 20 μM (C) for the indicated time. Representative results of four independent experiments are shown. Quantifications (bottom) of eIF2α phosphorylation in immunoblots such as shown above. Data are means ± SEM, n = 4. ^∗^p < 0.05, ^∗∗^p < 0.01, ^∗∗∗^p < 0.001 by unpaired two-tailed Student t test in comparison to 0 hr time point. ns, not significant. (D and E) Upper panel: Autoradiogram of newly synthesized proteins radiolabeled with ^35^S-methionine in HeLa cells treated with Raphin1 at 10 (D) or 20 μM (E) for the indicated time. Lower panel: Coomassie-stained gel. Representative results of three independent experiments are shown. (F) HeLa cells were plated in a 96-well plate and treated with indicated concentrations of Raphin1 in the presence of CellTox Green Dye (Promega). Cell confluency and green fluorescence (representing dead or dying cells) was measured as a function of time using the IncuCyte ZOOM system (Essen BioScience). Data is expressed as % of dead cells (described in the STAR Methods). Representative results of three independent experiments are shown. Each data point represents means ± SEM.

Because Raphin1 was stable over the duration of the treatment (Figure S4A), we wondered why 10 μM Raphin1 induced a transient increase in eIF2α phosphorylation, resulting in a transient decrease in protein synthesis (Figures 3A and 3B). We noted that R15A expression coincided with the translation recovery observed 10 hr after Raphin1 (10 μM) addition (Figures 3A and 3B), suggesting that R15A mediated eIF2α dephosphorylation and translation recovery in Raphin1-treated cells. This observation implies that Raphin1 at 10 μM selectively inhibited R15B, but not R15A, in cells, in agreement with the ∼30-fold selectivity of Raphin1 for R15B-PP1c, relative to R15A-PP1c, measured in the holophosphatase SPR assay (Figure 2C). The relative selectivity of Raphin1 for R15B over R15A is important because R15A is closely related to R15B. To assess the selectivity limit in cells, we treated cells at a higher concentration. In contrast to the 10 μM treatment, Raphin1 at 20 μM caused a persistent phosphorylation of eIF2α, resulting in a persistent inhibition of protein synthesis (Figures S4B–S4E), suggesting that at 20 μM, Raphin1 inhibited both R15B and R15A. Supporting this interpretation, Raphin1 was toxic at 20 μM (Figure S4F). Likewise, genetic inactivation of either R15A or R15B is viable in cells, but inactivation of the two eIF2α phosphatases is lethal (Harding et al., 2009). Therefore, subsequent experiments were conducted at 10 μM or below, at concentrations at which the compound is selective for R15B. To further validate this notion, we reasoned that the transient eIF2α phosphorylation and translation attenuation following R15B inhibition would be rendered persistent in the absence of R15A. Indeed, Raphin1-induced eIF2α phosphorylation and translation attenuation persisted in the presence of the R15A inhibitor GBZ (Figures 3C and 3D) or upon genetic inactivation of R15A (Figures 3E and 3F). Importantly, all the measurable effects of Raphin1 on eIF2α phosphorylation and translation were abolished in *R15b −/−* cells (Figures 3G and 3H). This demonstrates that the measured activity of Raphin1 in cells up to 10 μM is mediated by an on-target inhibition of R15B. Inhibition of R15B evokes a transient increase in the phosphorylation of eIF2α, resulting in a transient attenuation of protein synthesis (Figure 3I). These changes are transient because Raphin1 spares R15A, which mediates eIF2α dephosphorylation and translation recovery following R15B inhibition.

### Raphin1 Selectively Inhibits Recombinant R15B in Biochemical Assays

**Figure 4:**
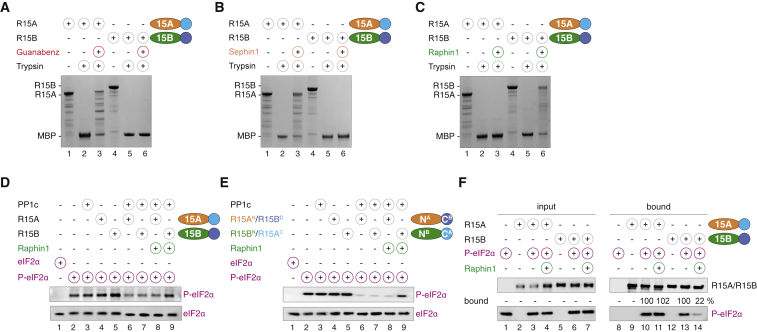
Raphin1 Inhibits Recombinant R15B (A–C) Coomassie-stained gels showing limited trypsin (5 nM) digestion of R15A and R15B in the presence of Guanabenz (A), Sephin1 (B), Raphin1 (C), or DMSO (vehicle) carried out for 5 min at 22°C. (D) Immunoblots of P-eIF2α and eIF2α following a dephosphorylation reaction by R15-PP1c holoenzymes in the presence of Raphin1. (E) Immunoblots of P-eIF2α and eIF2α following a dephosphorylation reaction by R15 chimera-PP1c holoenzymes in the presence of Raphin1. (F) Immunoblots of P-eIF2α binding to MBP-tagged R15B^340–698^ or R15A^325–636^ in the presence or absence of Raphin1. Representative results of three independent experiments are shown.

Next, we wanted to gain further insights into the mechanism of action of Raphin1 and used a set of recently developed biochemical assays that have enabled the functional characterization of R15A and its inhibitors (Carrara et al., 2017). As previously reported (Carrara et al., 2017), GBZ or Sephin1 induced a conformational change in the isolated R15A, which was detected by increased resistance to limited-proteolysis (Figures 4A and 4B). This activity was selective because neither GBZ nor Sephin1 affected R15B (Figures 4A and 4B), as previously reported (Carrara et al., 2017). In contrast, Raphin1 had no effect on the protease sensitivity of R15A but protected R15B (Figure 4C). This suggests that Raphin1 induces a conformational change in R15B. In addition, this confirms the selectivity of Raphin1 for R15B over R15A. To measure if this had functional consequences, we used the recombinant holophosphatases R15A-PP1c and R15B-PP1c in a biochemical activity assay previously described (Carrara et al., 2017). We found that Raphin1 inhibited dephosphorylation of eIF2α by R15B-PP1c, but not by R15A-PP1c (Figure 4D). Importantly, inhibition of eIF2α dephosphorylation by Raphin1 was transferable from R15B to R15A by swapping their amino-terminal regions (Figure 4E). Having previously found that the amino-terminal region of R15A or R15B is responsible for substrate binding (Carrara et al., 2017), we examined if Raphin1 affected this activity. We found that Raphin1 decreased the recruitment of eIF2α to R15B, but had no such effect on R15A (Figure 4F). Thus, Raphin1 selectively binds to R15B, leading to an alteration of its substrate recruitment function, thereby decreasing dephosphorylation.

### Raphin1 Renders R15B Prone to Degradation in a p97-Dependent Manner

**Figure 5:**
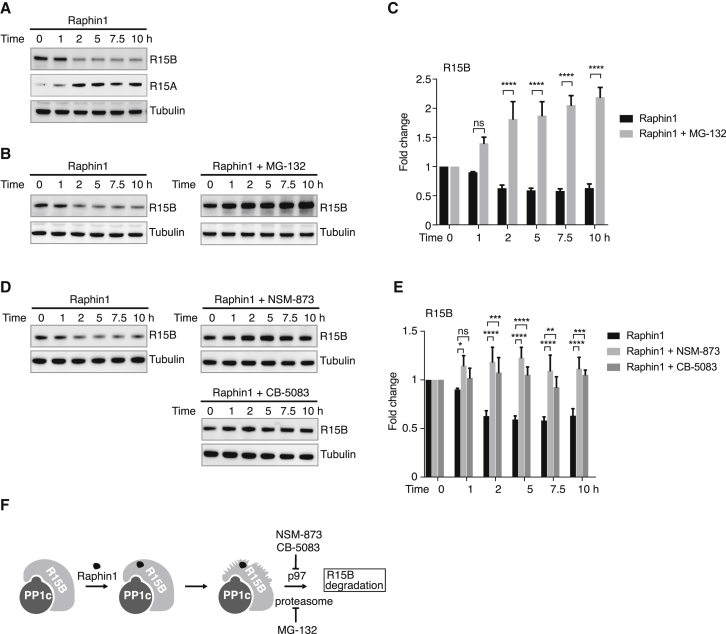
Raphin1 Promotes Proteasome- and p97-Dependent Degradation of R15B (A, B, and D) Immunoblots of the indicated proteins in HeLa cells lysates treated with Raphin1 at 10 μM, in the absence (A) or presence of the proteasome inhibitor MG-132 at 10 μM (B) or the p97 inhibitors NSM-873 or CB-5083 at 1 μM (D) for the indicated time. Representative results of three independent experiments are shown. (C) Quantifications of immunoblots corresponding to experiments shown in (B). Data are means ± SEM, ^∗∗∗∗^p < 0.0001 by two-way ANOVA. ns, not significant. (E) Quantifications of immunoblots corresponding to experiments shown in (D). Data are means ± SEM, n = 3. ^∗^p < 0.05; ^∗∗^p < 0.01; ^∗∗∗^p < 0.001; ^∗∗∗∗^p < 0.0001 by two-way ANOVA. ns, not significant. (F) A proposed model depicting Raphin1 mechanism of action: Raphin1 binds to R15B and induces a conformational change that in cells results in its degradation in a proteasome- and p97-dependent manner.

Having observed that Raphin1 induced a conformational change using recombinant R15B, we wondered what could be the consequences of such an event in a complex cellular environment. In cells, proteins of abnormal conformation are usually recognized by quality control systems and targeted to degradation (Pilla et al., 2017). Therefore, we next thought to assess the effect of Raphin1 on the levels of R15B. We found that R15B levels significantly decreased upon treatment with Raphin1 (Figure 5A). This was not observed for R15A (Figure 5A), further validating the selectivity of Raphin1 for R15B over R15A. The Raphin1-induced decrease in R15B was prevented by a co-treatment with the proteasome inhibitor MG-132 (Figures 5B and 5C). This finding suggests that Raphin1 induces a conformational change in R15B, which in cells, resulted in its degradation. R15B in cells is not free but bound to PP1c (Virshup and Shenolikar, 2009). Because the proteasome degrades single polypeptides, but not protein complexes, R15B ought to be freed from its interaction partner to be degraded. The AAA ATPase p97 is involved in a variety of cellular processes through its ability to extract and pull proteins out of membranes or protein complexes to deliver them to the proteasome (van den Boom and Meyer, 2018). Thus, we next tested if p97 was involved in the degradation of R15B followed by Raphin1 treatment using two different p97 inhibitors, NSM-873 and CB-5083 (Deshaies, 2014, Nguyen et al., 2017). We found that NSM-873 and CB-5083 essentially abrogated the decrease in R15B abundance caused by Raphin1 (Figures 5D and 5E). While this manuscript was under review, R15B was reported to be a substrate of p97 (Hülsmann et al., 2018). The model emerging from the findings presented here is as follows: Raphin1 binds to R15B and induces a conformational change that targets it to the proteasome for degradation in a p97-dependent manner (Figure 5F).

### Raphin1 Is Suitable for *In Vivo* Studies

**Figure S5:**
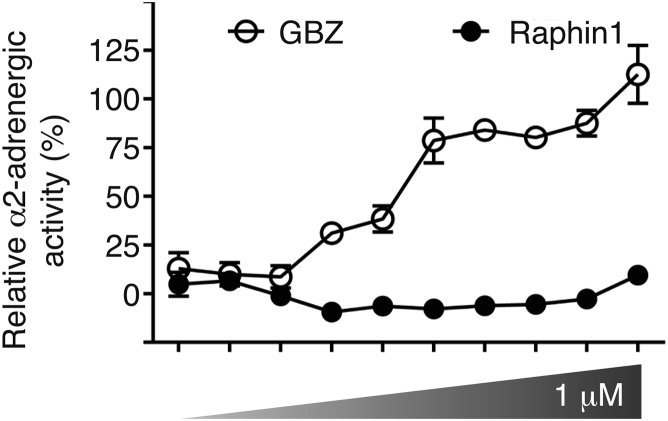
Raphin1 Has No Measurable Adrenergic Activity in Cells, Related to Figure 6 α-2 adrenergic activity measurement in cells expressing human recombinant receptor following GBZ or Raphin1 treatment. Data are means ± SEM, n = 3 per group.

**Figure 6:**
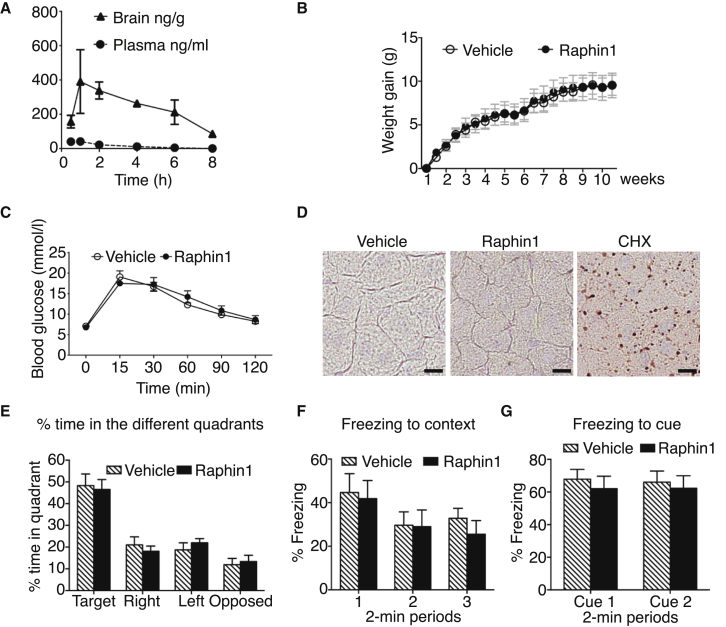
No Adverse Effects of Raphin1 Treatment on Body Weight Gain, Pancreatic and Liver Function, or Memory in Mice (A) Concentration of Raphin1 in the brain and plasma at the indicated time after a single oral administration of Raphin1 at 2 mg/kg. Data are means ± SEM; n = 3. (B) Total body weight gain of wild-type mice treated with Raphin1 at 2 mg/kg or vehicle for 10 weeks. Data are means ± SEM; n = 10 per group. (C) Glucose tolerance test on wild-type mice after treatment with Raphin1 at 2 mg/kg or vehicle for 8 weeks. Data are means ± SEM; n = 8 (vehicle) and n = 9 (Raphin1). (D) Oil Red O staining of liver in wild-type mice treated with Raphin1 or CHX at 40 mg/kg or vehicle. Scale bar, 10 μm. (E) Quadrant occupancy after training and removal of the platform in the Morris water maze of wild-type mice treated with Raphin1 at 2 mg/kg or vehicle for 2 weeks. Data are means ± SEM, n = 9 (vehicle) and n = 10 (Raphin1). (F and G) Response to fear conditioning—freezing to context (F) or freezing to cue (G)—of wild-type mice treated with Raphin1 at 2 mg/kg or vehicle for 3 weeks. Data are means ± SEM; n = 10 per group. There were no significant differences between Raphin1- and vehicle-treated mice as revealed by an unpaired two-tailed Student’s t test (B, C, E, F, and G). See also Figures S5 and S6 and Videos S1, S2, and S3.

**Figure S6:**
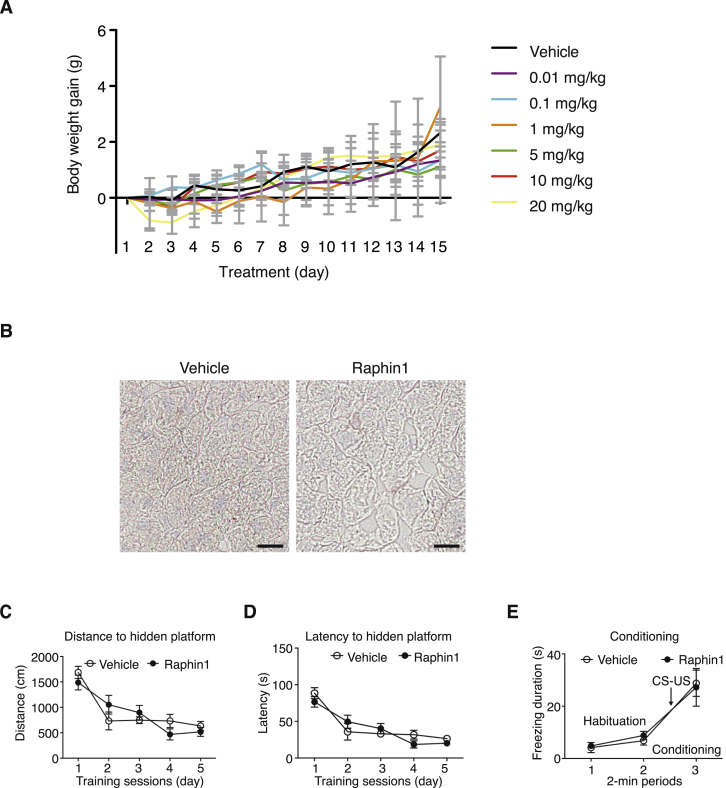
Raphin1 Has No Adverse Effect on Body Weight Gain in Mice, Does Not Cause Liver Steatosis, or Affects Memory, Related to Figure 6 (A) Body weight gain of wild-type mice treated with Raphin1 at the indicated concentration or vehicle for 15 days. Data are means ± SEM, n = 3 per group. (B) Oil Red O staining of liver in wild-type mice treated with vehicle or Raphin1 at 2 mg/kg for 10 weeks. Scale bar, 10 μm. (C and D) Performance in the learning phase of Morris Water Maze of mice treated with Raphin1 at 2 mg/kg or vehicle for 2 weeks. Data are means ± SEM, n = 9 (vehicle) or n = 10 (Raphin1). Parameters measured: Distance (C) and latency (D) to locate a hidden platform in training sessions for 5 consecutive days. (E) Performance in the conditioning phase of fear conditioning in mice treated with Raphin1 at 2 mg/kg or vehicle for 3 weeks. Data are means ± SEM, n = 10 per group. Parameter measured: Freezing response during the conditioning session, where a light/tone [conditioned stimulus – CS] and a foot shock [aversive unconditioned stimulus – US] were applied. There were no significant differences between Raphin1 and vehicle treated mice as revealed by the unpaired two-tailed Student t test (A, C, D, E).

Next, we examined if Raphin1 had properties compatible with *in vivo* studies. Because translation inhibitors, such as cycloheximide, are toxic, we first examined whether Raphin1 was safe. Raphin1 lacked the undesirable α2-adrenergic activity of GBZ (Figure S5; Videos S1, S2, and S3). Pharmacokinetic analysis of Raphin1, administered orally at 2 mg/kg, revealed that the molecule crossed the blood-brain barrier and concentrated in the brain with a peak concentration of ∼1.5 μM and a half-life of ∼4-6 hr (Figure 6A). We selected this dose for efficacy studies because this corresponds to a concentration where the compound was not toxic when continuously applied to cells for 48 hr (Figure S4F). Knowing that a total lack of R15B activity is detrimental during mammalian development (Abdulkarim et al., 2015, Harding et al., 2009) but reduction of R15B with small interfering RNA (siRNA) improves stress survival (Jousse et al., 2003), we dosed Raphin1 once a day reasoning that the half-life of Raphin1, ∼4–6 hr (Figure 6A), would ensure pulse inhibition of R15B and thereby reduce the risk of undesirable side effects. Unlike the complete loss of R15B activity (Abdulkarim et al., 2015, Harding et al., 2009), chronic treatment with Raphin1 at various doses once a day had no obvious adverse effects on weight gain (Figures 6B and S6A) or glucose tolerance (Figure 6C). In addition, in contrast to cycloheximide, which caused liver steatosis (Figure 6D) as expected (Jazcilevich and Villa-Treviño, 1970), Raphin1 caused no such dysfunction (Figures 6D and S6B). Phosphorylation of eIF2α affects memory (Costa-Mattioli et al., 2007). Chronic treatment with Raphin1 had no adverse effects on memory in the Morris water maze or in a fear-conditioning paradigm (Figures 6E–6G and S6C–S6E). This demonstrates that Raphin1 has no measurable adverse effects on weight, liver, and pancreatic function, or memory in diverse experimental paradigms.

Video S1. Video Showing Wild Type Mice before Treatment, Related to Figure 6

Video S2. Video Showing Wild Type Mice 15 Min after Treatment with GBZ or Raphin1 (10 mg/kg), Related to Figure 6

Video S3. Video Showing Wild Type Mice 30 Min after Treatment with GBZ or Raphin1 (10 mg/kg), Related to Figure 6

### Raphin1 Is Beneficial in Mutant Huntingtin Transgenic Mice

**Figure 7:**
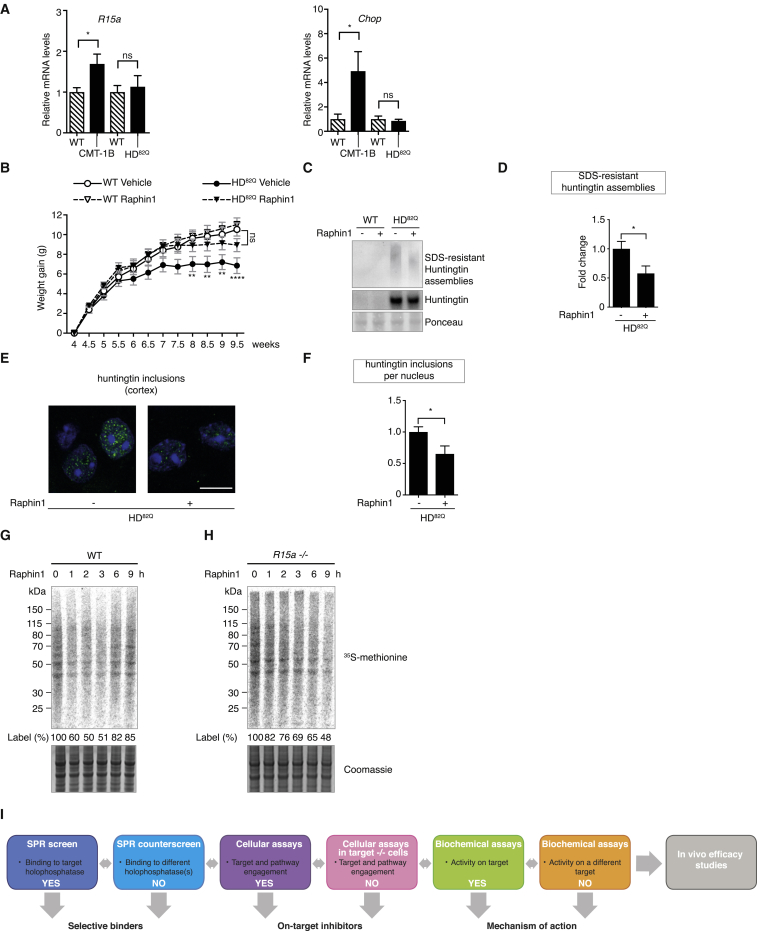
Raphin1 Normalizes Weight and Reduces Accumulation of SDS-Resistant Huntingtin Assemblies and Inclusions in Mutant Huntingtin Transgenic Mice (A) *R15a* and *Chop* mRNA levels (qPCR) in affected tissues from symptomatic CMT-1B or symptomatic HD^82Q^ mice relative to their wild-type littermates. Data are means ± SEM; n = 5 (CMT-1B) and n = 6 (HD^82Q^). ^∗^p < 0.05 by unpaired two-tailed Student’s t test. ns, not significant. (B) Body weight gain of wild-type and HD^82Q^ mice treated with Raphin1 at 2 mg/kg or vehicle once a day. Data are means ± SEM, n = 22, 24, 23, and 21 for WT vehicle, WT Raphin1, HD^82Q^ vehicle, and HD^82Q^ Raphin1, respectively. ^∗∗^p < 0.01; ^∗∗∗∗^p < 0.0001 by two-way ANOVA with Tukey’s multiple comparisons test. ns, not significant. (C) Immunoblots of cortex lysates of 2-month-old wild-type and HD^82Q^ mice treated with Raphin1 at 2 mg/kg or vehicle from 4 weeks of age daily. 2B4 antibody revealed SDS-resistant huntingtin assemblies, and 1C2 detected SDS-soluble huntingtin. (D) Quantifications of huntingtin SDS-resistant assemblies from immunoblots such as (C) from 2-month-old mice following treatment with Raphin1 or vehicle from 4 weeks of age. Data are means ± SEM; n = 6. ^∗^p < 0.05 by an unpaired two-tailed Student t’s test. (E) Representative images of nuclear huntingtin inclusions revealed with 2B4 antibody (green) in the cortex of 2-month-old HD^82Q^ mice treated with Raphin1 at 2 mg/kg or vehicle from 4 weeks of age daily. Nuclei, Hoechst 33342 (blue). Scale bar, 10 μm. (F) Quantification of nuclear huntingtin inclusions (see STAR Methods) revealed with 2B4 antibody in the cortex of 2-month-old mice following treatment with Raphin1 or vehicle from 4 weeks of age. Data are means ± SEM; n = 6. ^∗^p < 0.05 by an unpaired two-tailed Student’s t test. (G and H) Upper panel: autoradiogram of newly synthesized proteins radiolabelled with 75% ^35^S-methionine + 25% ^35^S-cysteine in brains of wild-type (G) or *R15a −/−* (H) mice treated with Raphin1 (40 mg/kg) for the indicated time. Lower panel: Coomassie-stained gel. (I) Cartoon depicting the platform of assays to enable the target-based discovery of holophosphatase inhibitors. A platform of biophysical, biochemical, and cell-based assays was used to identify selective holophosphatase inhibitors. An SPR screen identifies compounds (hits) binding to a target holophosphatase and an SPR counter screen performed with a different holophosphotase filters for selective binders. For selectivity, a filter of dissociation constant of at least 5-fold for one holophosphatase over the other is set. Hits from the primary binding screen are validated in cellular assays measuring target and pathway engagement. On-target inhibitors (lead) lose activity in cells knocked out for the target. Biochemical assays (optional) examine the mechanism of action and further confirm selectivity of the compounds. *In vivo* efficacy studies were carried out with validated compounds. See also Figure S7.

**Figure S7:**
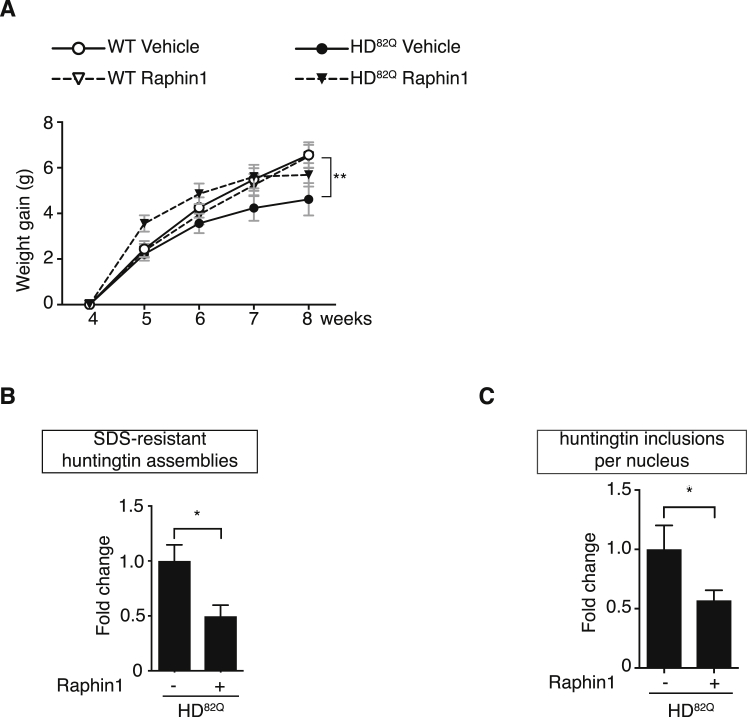
Raphin1 Is Beneficial in HD^82Q^ Mice, Related to Figure 7 Note that the results shown here were obtained with a separate cohort than the ones shown in Figures 7B, 7D, and 7F. (A) Total body weight gain of wild-type and HD^82Q^ mice treated orally with Raphin1 at 2 mg/kg or vehicle once a day for four weeks daily. Data are means ± SEM, n = 27, 26, 19, 21 for WT Vehicle, WT Raphin1, HD^82Q^ Vehicle and HD^82Q^ Raphin1, respectively. ^∗∗^p < 0.01 by two-way ANOVA with Tukey’s multiple comparisons test. (B) Quantifications of huntingtin assemblies from immunoblots such as (Figure 7C) performed on cortex lysates from 2.5-month-old mice following treatment with Raphin1 or vehicle from 4 weeks of age. Data are means ± SEM, n = 3. ^∗^p < 0.05 by unpaired two-tailed Student t test. (C) Quantifications of nuclear huntingtin inclusions (see STAR Methods) revealed with 2B4 antibody in the cortex of 2.5-month-old mice following treatment with Raphin1 or vehicle from 4 weeks of age. Data are means ± SEM, n = 7 (vehicle) and n = 10 (Raphin1). ^∗^p < 0.05 by unpaired two-tailed Student t test.

Abnormal folding of proteins is at the origin of a broad range of diseases (Powers et al., 2009). The R15A inhibitor Sephin1 is a proteostatic enhancer found to be efficacious in two models of neurodegenerative diseases in mice associated with ER stress: Charcot-Marie-Tooth 1B (CMT-1B) and SOD1-ALS (Das et al., 2015, Tsaytler et al., 2011). Because R15A is stress inducible, the use of R15A inhibitors is restricted to conditions inducing R15A (Bertolotti, 2018). ER stress has been reported in a variety of cells overexpressing a fragment of mutant huntingtin, the protein associated with Huntington’s disease (HD) (Duennwald et al., 2006, Lajoie and Snapp, 2011, Leitman et al., 2013). However, it is unclear if ER stress is associated with HD *in vivo*. Conflicting results have been reported in transgenic mouse models with some observing no global ER stress using a variety of markers (Vidal et al., 2012), while others have suggested ER stress induction based on immunohistochemistry experiments (Leitman et al., 2014). These differences could be attributed to the difficulties in reliably detecting ER stress *in vivo*. We therefore used the sensitive and quantitative qPCR readouts we previously reported (Das et al., 2015) to examine whether the ER stress-inducible *R15a* was increased in symptomatic N171-82Q mice (HD^82Q^), a model of HD. As previously reported (Das et al., 2015), *R15a* was increased in affected tissues from symptomatic CMT-1B mice relative to wild-type (WT) controls. In contrast, *R15a* levels were similar to those in wild-type in cortex from symptomatic HD^82Q^ mice (Figure 7A). To confirm this, we used another sensitive ER stress marker, *Chop,* knowing that *Chop* and *R15a* are in the same pathway. *Chop* was highly induced in affected tissues from symptomatic CMT-1B mice, but not from symptomatic HD^82Q^ mice (Figure 7A). The absence of induction of R15A in HD^82Q^ mice implied that R15A is not a therapeutic target for this condition. In contrast to R15A, R15B is constitutively expressed (Jousse et al., 2003). Thus, we used HD^82Q^ mice to test the potential benefit of R15B inhibition and treated HD^82Q^ mice with Raphin1. HD^82Q^ mice lose weight over time and accumulate SDS-insoluble mutant huntingtin assemblies (Schilling et al., 1999). Raphin1 improved weight of HD^82Q^ mice treated from 4 to ∼10 weeks of age with 2 mg/kg of Raphin1 once a day by oral gavage (Figure 7B). Raphin1 also decreased SDS-insoluble huntingtin assemblies (Figures 7C and 7D) and nuclear inclusions in the cortex of HD^82Q^ mice (Figures 7E and 7F). Attesting the robustness of the treatment, a similar benefit of Raphin1 treatment was observed in a separate cohort on body weight as well as SDS-insoluble assemblies and nuclear inclusions (Figure S7). Monitoring dynamic phosphorylation events *in vivo* is not trivial because minute changes are sufficient to elicit biological changes. We therefore aimed at measuring protein synthesis to monitor Raphin1 activity *in vivo*. As in cells (Figure 3B), we observed that Raphin1 transiently reduced protein synthesis in the brains of wild-type mice (Figure 7G). Translation recovery was observed ∼6 hr after Raphin1 administration (Figure 7G) in wild-type brains but this recovery was reduced in the brains of *R15a −/−* mice (Figure 7H). This demonstrates that *in vivo* as in cells, Raphin1 induces a transient attenuation of protein synthesis and translation recovery following Raphin1 treatment depends on R15A.

## Discussion

Here, we have developed a platform that enabled the first target-based discovery of an inhibitor of a regulatory subunit of the PP1 phosphatase. Raphin1 inhibits R15B, by inducing a conformational change, compromising substrate recruitment and dephosphorylation. The Raphin1-induced conformational change renders R15B prone to degradation, in a p97- and proteasome-dependent manner. In cells and *in vivo*, selective inhibition of R15B by Raphin1 resulted in transient attenuation of protein synthesis, the reversibility of eIF2α phosphorylation being ensured by R15A-PP1. Raphin1 is potent, is orally available, crosses the blood-brain barrier, and reduces organismal and molecular deficits in a mouse model of a protein misfolding disease. This identifies R15B as a druggable target for protein misfolding conditions and establishes a platform for target-based discovery of a regulatory subunit of serine/threonine phosphatase (Figure 7I).

Here, we show the benefit of Raphin1 in a mouse model of HD, which was selected as an example of a neurodegenerative condition caused by the misfolding of a protein. HD is a monogenic disorder due to a dominantly inherited mutation in huntingtin (The Huntington’s Disease Collaborative Research Group, 1993). Unlike other neurodegenerative diseases, such as Alzheimer’s or Parkinson’s diseases, which are mostly sporadic (Singleton and Hardy, 2016), HD is exclusively familial. With pre-symptomatic genetic testing being available, HD patients can be selected before the disease is too far advanced for intervention. From that point of view, the treatments applied to the HD mice mimic this scenario.

The discovery of Raphin1 in a library of GBZ derivatives showed that altering the positions of the chloro-substituents on the benzyl ring switches the selectivity of these molecules from R15A to R15B. Because the founding principle of medicinal chemistry is that structurally similar molecules hit the same target with similar properties (Martin et al., 2002), the fact that GBZ and Raphin1 are two isomers with different pharmacological properties may appear, at first glance surprising. However, a second and prevalent principle of medicinal chemistry is that discrete changes in the structure of drug molecules often induce potent changes in pharmacological profile (Kubinyi, 1998, Wermuth, 2006). Abundant examples exist to document this second principle, particularly in the field of G-protein-coupled receptor (GPCR) drugs. For example, similar to what we report here, where a subtle change in one substituent of GBZ changes its selectivity from R15A to R15B, the functional response for angiotensin AT1 and AT2 receptors is switched upon minimal chemical variation of an agonist (Fujioka and Omori, 2012). Like R15A and R15B, AT1 and AT2 share low (<30%) sequence similarity (Fujioka and Omori, 2012). Although the research on selective phosphatase inhibitors is in its infancy, the analogy between selective inhibitors of R15 and GPCR drugs provides a conceptual framework to further develop phosphatase drug discovery.

The reversible phosphorylation of proteins is one of the most prevalent and ubiquitous modes of regulation, controlling virtually all biological processes through the opposite action of protein kinases and phosphatases. Kinases are prevalent drug targets, with more than 3,000 approved and experimental drugs (Rask-Andersen et al., 2014), unlike phosphatases which have been traditionally overlooked. There are about 200 PP1 holophosphatases in mammals, which are heteromeric holoenzymes sharing the same catalytic subunit PP1c bound to one or two of at least 200 diverse regulatory subunits (Bollen et al., 2010, Virshup and Shenolikar, 2009). Because PP1c is a catalytic subunit shared by hundreds of holoenzymes, inhibitors of PP1c such as Calyculin A are not selective and highly toxic (McCluskey et al., 2002). The selective inhibitors of the regulatory phosphatase R15A, GBZ and Sephin1, were discovered through phenotypic screens, leading to the realization that selective inhibition of a holophosphatase can be achieved by targeting its regulatory subunit (Das et al., 2015, Tsaytler et al., 2011). While the same paradigm could be in principle applicable to other holoenzymes, the generalization of this concept was hindered by the lack of screening methods to enable the target-based discovery of inhibitors of regulatory subunits of phosphatases.

Here, we overcame this challenge and developed a platform, comprising a suite of biophysical and cellular screens and counter screens (Figure 7I), that led to the identification of a first inhibitor of a selected target, R15B. The method provided here is versatile and can be applied to enable target-based drug discovery to diverse regulatory subunits of phosphatases, and more generally to multi-protein complexes. This work provides the conceptual and methodological framework to exploit regulatory subunits of phosphatases as drug targets, opening up opportunities to identify small-molecule inhibitors and manipulate cell function, perhaps for therapeutic benefits.

## STAR★Methods

### Key Resources Table

**Table:** 

REAGENT or RESOURCE	SOURCE	IDENTIFIER
**Antibodies**		

Pierce High Sensitivity Streptavidin-HRP antibody	ThermoFisher Scientific	Cat#21130
Rabbit polyclonal e-IF2α-P antibody	ThermoFisher Scientific	Cat#44-728G; RRID: AB_2533736
Mouse monoclonal e-IF2α antibody	Abcam	Cat#ab5369; RRID: AB_304838
Mouse monoclonal tubulin antibody	Sigma-Aldrich	Cat#T5168; RRID: AB_477579
Mouse monoclonal BiP antibody	BD Biosciences PharMingen	Cat#610978; RRID: AB_398291
Rabbit polyclonal ATF4 antibody	Santa Cruz Biotechnology	Cat#sc-200; RRID: AB_2058752
Rabbit polyclonal Ppp1r15a antibody	Proteintech	Cat#10449-1-AP; RRID: AB_2168724
Rabbit polyclonal Ppp1r15b antibody	Proteintech	Cat# 14634-1-AP; RRID: AB_2300036
Mouse monoclonal CHOP antibody	ABR Affinity BioReagents	Cat#MA1-250; RRID: AB_2292611
Mouse monoclonal huntingtin antibody 2B4 antibody	Euromedex	Cat#HU-2B4
Mouse monoclonal huntingtin antibody 1C2 antibody	Euromedex	Cat#PQ-1C2
Goat polyclonal Alexa Fluor 488 antibody	ThermoFisher Scientific	Cat#A11001; RRID: AB_2534069
Mouse monoclonal MBP-HRP antibody	New England BioLabs	Cat#E8038; RRID: AB_1559738
Rabbit polyclonal e-IF2α antibody	Abcam	Cat#ab26197; RRID: AB_2096478
Rabbit polyclonal e-IF2α-P (Ser51) antibody	Cell Signaling Technology	Cat#9721; RRID: AB_330951

**Bacterial and Virus Strains**		

*E. coli* BL21-GOLD (DE3) pLysS	Agilent Technologies	Cat#230134
*E. coli* BL21/pGro7	Takara	Cat#9122

**Chemicals, Peptides, and Recombinant Proteins**		

MBP-R15A^325-636^-His (R15A)	Das et al., 2015	N/A
MBP-R15B^340-698^-His (R15B)	Das et al., 2015	N/A
bio-PP1c^1-322^ (bio-PP1c)	This study	N/A
his-PP1c^1-322^ (his-PP1c)	This study	N/A
MBP-R15A^325-512^R15B^636-698^-His (R15A^N^-R15B^C^)	Carrara et al., 2017	N/A
MBP-R15B^340-635^R15A^513-636^-His (R15B^N^-R15A^C^)	Carrara et al., 2017	N/A
PP1c^7-330^ (PP1c)	Carrara et al., 2017	N/A
GST-PERK	Carrara et al., 2017	N/A
eIF2a^1-185^-His	Carrara et al., 2017	N/A
bio-GBZ	Tsaytler et al., 2011	N/A
Guanabenz (GBZ)	Sigma-Aldrich	Cat#G110
Sephin1	Das et al., 2015	N/A
Raphin1	This study	N/A
Compound C3	This study	N/A
MG-132	Cell Signaling technology	Cat#2194
NSM-873	Selleckchem	Cat# S7285
CB-5083	Cayman Chemicals	Cat#2194
CalyculinA	Cell signaling technology	Cat#9902S
CellTox Green Dye, 1,000X	Promega	Cat#G873B
Cycloheximide	Sigma-Aldrich	Cat#C7698
Hematoxylin	VWR International	Cat#351945S
Oil Red O solution	VWR International	Cat#101410-976
RNeasy Mini Kit	QIAGEN	Cat#74104
iScript cDNA Synthesis Kit	Bio-Rad	Cat#1708891
SYBR Select Master Mix	Applied Biosystems	Cat#44-729-08
O.C.T. compound	VWR International	Cat#361603E
Hoechst 33342	Lonza	Cat#PA-3014

**Critical Commercial Assays**		

Bac-to-Bac Baculovirus Expression System	ThermoFisher Scientific	Cat#10359-016
EnzChek Phosphatase Assay Kit	ThermoFisher Scientific	Cat#E12020
Accu-Chek Aviva Blood Glucose Meter	Roche	Cat#06351557018
Accu-Chek Aviva Glucose Test Strips	Roche	Cat#06453970

**Experimental Models: Cell Lines**		

HeLa (Female)	Sigma-Aldrich	IGBMC, Illkirch, France
MEF cells: *Ppp1r15a −/−* (*R15a −/−*). Sex undetermined.	Harding et al., 2009	N/A
MEF cells: *Ppp1r15b −/−* (*R15b −/−*) Sex undetermined.	Harding et al., 2009	N/A
*S. frugiperda* Sf9 SFM adapted insect cells. Sex undetermined.	ThermoFisher Scientific	Cat#11496015

**Experimental Models: Organisms/Strains**		

C57BL/6J	The Jackson Laboratory	Cat#000664
B6C3-Tg(HD82Gln)81Gschi/J (N171-82Q)	The Jackson Laboratory	Cat#003627
CMT-1B	Lawrence Wrabetz	N/A
B6.129P2-Ppp1r15atm1.1Ajf/Mmnc (R15a −/−)	MMRRC (mutant mouse resource and research centers)	Cat#30266

**Oligonucleotides**		

*R15a* – f: GACCCCTCCAACTCTCCTTC	Sigma-Aldrich	N/A
*R15a* – r: TCTCAGGTCCTCCTTCCTCA	Sigma-Aldrich	N/A
*Chop* – f: GGAGAGAGTGTTCAAGAAGGAAGTG	Sigma-Aldrich	N/A
*Chop* – r: GCAGGTCCTCATACCAGGCTT	Sigma-Aldrich	N/A
*Gapdh* – f: TGGGTGGTCCAGGGTTTCTTACTCCTT	Sigma-Aldrich	N/A
*Gapdh* – r: CGACTTCAACAGCAACTCCCACTCTTCC	Sigma-Aldrich	N/A

**Recombinant DNA**		

pMAL-c5x	New England BioLabs	Cat#8108S
Bac-to-Bac vector kit (pFastBac1)	ThermoFisher Scientific	Cat#10360014
pDW464	Addgene	Cat#8845
Plasmid pMAL-c5x-R15A^325-636^ (N-terminal MBP-tag, C-terminal 6 × His-tag)	Das et al., 2015	N/A
Plasmid pMAL-c5x-R15B^340-698^ (N-terminal MBP-tag, C-terminal 6 × His-tag)	Das et al., 2015	N/A
Plasmid pDW464-PP1γc^1-322^ (N-terminal BAP-tag)	This study	N/A
Plasmid pFastBac1-6 × His-PP1γc^1-322^ (N-terminal 6 × His-tag)	This study	N/A
Plasmid pMAL-c5x-R15A^325-512^R15B^636-698^ (N-terminal MBP-tag, C-terminal 6 × His-tag)	Carrara et al., 2017	N/A
Plasmid pMAL-c5x-R15B^340-635^R15A^513-636^ (N-terminal MBP-tag, C-terminal 6 × His-tag)	Carrara et al., 2017	N/A
Plasmid modified pGEX6p1-PP1αc^7-330^ (the vector's GST-tag was replaced by an N-terminal Thio6/His6-tag (MGSDKIHHHHHH)).	Carrara et al., 2017	N/A
Plasmid pGEX-4T-1-PERK^537-1114^ (N-terminal GST-tagged murine PERK kinase domain)	Addgene	Cat#21817
Solubility enhanced human eIF2a (amino acids 1-185) with a C-terminal His-tag	Ito et al., 2004	N/A

**Software and Algorithms**		

Biacore T200 Evaluation Software	GE Healthcare	https://www.biacore.com/lifesciences/service/downloads/software_licenses/biaevaluation/
GraphPad Prism 7	GraphPad Software	https://www.graphpad.com/scientific-software/prism/
ImageJ	NIH	https://imagej.nih.gov/ij/
IncuCyte Zoom software	Essen BioScience	https://www.essenbioscience.com/en/
ViewPoint	ViewPoint Behavior Technology, France	http://www.viewpoint.fr/en/home
Coulbourn Instruments	Coulbourn Instruments, Allentown, PA, USA	https://www.coulbourn.com/
Graphic State software	Coulbourn Instruments, Allentown, PA, USA	https://www.coulbourn.com/
NIS Elements	Nikon Instruments	https://www.nikoninstruments.com/en_EU/Products/Software

### Contact for Reagent and Resource Sharing

Further information and requests for reagents should be directed to and will be fulfilled by the Lead Contact, Anne Bertolotti (aberto@mrc-lmb.cam.ac.uk).

### Experimental Model and Subject Details

#### Animal procedures

##### Ethical statement on mouse studies

All animal care and procedures were performed in compliance with the regulation on the use of Animals in Research (UK Animals Scientific Procedures Act of 1986 and the EU Directive 2010/63/EU) under the project license number 70/7956 with local ethical approval form the LMB Animal Welfare and Ethical Review committee.

##### Housing and husbandry of experimental animals

Animals were housed and cared for according to the Home Office Code of Practice for the Housing and Care of Animals used in Scientific Procedures. The ARRIVE guidelines have been followed in reporting this study (Kilkenny et al., 2010).

All animal experiments were undertaken with the approval of the Home Office, UK. Mice were kept in specific pathogen free ventilated cages (Tecniplast GM500, Tecniplast) on Lignocel FS14 spruce bedding (IPS) and Enviro-Dri nesting material (LBS) at 19-23°C with 12 hr light dark cycle with light from 7.00 am to 7.00 pm. The experimental animals were fed with Dietex CRM pellets (Special Diet Services). Maximum number of mice housed in individual cage was 2-3 per cage and occasionally 4 to homogenize the housing conditions. To monitor health conditions, all experimental animals were visually checked every day, cleaned out when soiled and a physical health check was performed each week on all mice. The experimental animals were weighed weekly, bi-weekly or daily. The experimental animals reaching moderate severity limit were provided with mash (Dietex CRM pellets soaked in water).

4-14 week-old C57BL/6J males and females (The Jackson Laboratory, stock 000664), 4-12 week-old N171-82Q (HD^82Q^) males maintained in mixed background (C3H/B6) (The Jackson Laboratory, stock 003627), 6 month-old CMT-1B males and females (Wrabetz et al., 2006), transgenic for mutant myelin protein zero - with deletion of serine 63 (P0S63del) maintained in the FVB/N background and 14 week-old B6.129P2-*Ppp1r15a*^*tm1.1Ajf*^/Mmnc (*R15a −/−*) males (mutant mouse resource and research centers - MMRRC) maintained in the C57BL/6J background were used. Littermates were allocated into experimental groups randomly. Where both genders were used there were no phenotypic differences between genders observed.

#### Cell lines

Mammalian cells were all grown in a humidified incubator with 5% CO_2_ at 37°C. HeLa cells were maintained in Dulbecco’s Modified Eagle’s Media (DMEM, ThermoFisher Scientific) supplemented with 100 U/mL penicillin and 100 μg/mL streptomycin (ThermoFisher Scientific), 2 mM L-glutamine (ThermoFisher Scientific) and 10% fetal bovine serum (FBS, ThermoFisher Scientific). *Ppp1r15a −/−* (*R15a −/−*) and *Ppp1r15b −/−* (*R15b −/−*) MEF cells (Harding et al., 2009) were maintained in DMEM (ThermoFisher Scientific) supplemented with 100 U/mL penicillin and 100 μg/mL streptomycin (ThermoFisher Scientific), 2 mM L-glutamine (ThermoFisher Scientific), 55 μM β-mercaptoethanol, 1X non-essential amino acids (Sigma-Aldrich) and 10% FBS (ThermoFisher Scientific). Cells were authenticated using PCR and western blotting.

#### Bacterial strains

Regulatory subunits and MBP for SPR studies were expressed in BL21-Gold (DE3) pLysS *E. coli* cells (Agilent Technologies) pre-cultured at 37°C and then shifted to 30°C for induction in 2 x TY medium for 3 hr. Other regulatory subunits, GST-PERK and eIF2a were expressed in BL21-Gold (DE3) pLysS *E. coli* cells (Agilent Technologies) pre-cultured at 37°C and then shifted to 30°C for induction in Luria Broth (LB) medium overnight (regulatory subunits) or for 6 hr. PP1 was expressed in BL21/pGro7 cells (Takara) pre-cultured at 35°C and then shifted to 10°C for induction in LB medium supplemented with 50 μg/mL ampicillin, 35 μg/mL chloramphenicol and 2 mM MnCl_2_.

#### Insect cells

*Spodoptere frugiperda* Sf9 SFM adapted cells (ThermoFisher Scientific) were propagated at 27°C in Insect-Xpress media (Lonza). For his-PP1c and bio-PP1c protein expression, stably transfected Sf9 cell pools were expanded and grown at 27°C in Insect-Xpress media (Lonza).

### Method Details

#### Protein expression and purification

As described in Das et al. (2015) the cDNAs encoding amino acids 325-636 of R15A and 340-698 of R15B were cloned into pMAL-c5x (New England BioLabs) for expression of N-terminal maltose-binding protein (MBP) fusion proteins. Additionally, a C-terminal hexahistidine-tag was added to the constructs. Empty pMAL-c5x vector, encoding for MBP protein, was used as a control where specified. Recombinant MBP-R15A^325-636^-His (R15A) and MBP-R15B^340-698^-His (R15B) were expressed in BL21-Gold (DE3) pLysS *E. coli* cells (Agilent Technologies). 80 mL of overnight culture was added to 800 mL of 2 x TY medium containing ampicillin (100 mg/L) and cells were grown to an optical density of OD_600_ = 0.6 in an incubator/shaker at 37°C and 210 revolutions per minute (rpm). Protein expression was induced by adding 0.1 M IPTG to the culture and growing for another 3 hr at 30°C. Cells were harvested by centrifuging at 4,500 rpm for 15 min and the pellets resuspended in PBS pH 7.4, 1 complete protease inhibitor cocktail tablet (PiC, Roche) per 50 mL and 0.2 mM PMSF. The resuspended pellets were flash frozen in liquid nitrogen and stored at −80°C until purification. The cells were thawed slowly on ice and the cells were lysed by sonication at 4°C. The bacterial lysates were cleared by centrifugation at 35,000 × g at 4°C for 60 min, filtered through a 0.45 μm filter and loaded onto a 5 mL HisTrap HP column (GE Healthcare) pre-equilibrated with PBS pH 7.4, 20 mM imidazole. After washing with 10 column volumes (CV) PBS pH 7.4, 20 mM imidazole, proteins were eluted with 8 CV PBS pH 7.4, 250 mM imidazole. The eluate was loaded onto a 5 mL MBPTrap HP column (GE Healthcare) pre-equilibrated in PBS pH 7.4. After washing with 5 CV PBS pH 7.4, proteins were eluted with 5 CV PBS pH 7.4, 20 mM maltose. The proteins were analyzed on Bolt SDS-PAGE 4%–12% Bis-Tris gels (ThermoFisher Scientific) stained with InstantBlue (Expedeon). Samples containing pure R15A or R15B were pooled, dialysed against PBS pH 7.4, concentrated and stored at −80°C in small aliquots.

cDNA encoding for human PP1cγ (aa 1-322) was cloned into two baculovirus transfer vectors for expression in insect cells; pFastBac1 (ThermoFisher Scientific) with a N-terminal His_6_ tag to generate his-PP1c and pDW464 (Addgene) to add a N-terminal biotin acceptor peptide (BAP) to generate biotinylated PP1c (bio-PP1c). The pDW464 vector encodes for the *E. coli* biotin holoenzyme synthetase (BirA), so that BAP-tagged proteins can be biotinylated *in vivo* in Spodoptere frugiperda (Sf9) insect cells (Duffy et al., 1998). The Bac-to-Bac baculovirus expression system (ThermoFisher Scientific) was used to generate the two recombinant bacmid DNAs and Sf9 insect cells were used to amplify the viral stocks. Protein expression was induced by infection with the appropriate viral stock of 4 L of Sf9 insect cells in Insect-Xpress media (Lonza) at a density of 0.8 × 10^6^ cells/mL. The cells were harvested after 72 hr by centrifuging at 1,200 × g for 15 min. The pellets were washed with PBS and transferred to 50 mL falcon tubes. The cells were lysed in lysis buffer (50 mM Tris pH 7.4, 150 mM NaCl, 0.2% triton, 5% glycerol, 1 PiC tablet (Roche) per 50 ml, 0.2 mM PMSF) at 4°C on a rotary wheel for 20 min. The lysed cells were flash frozen in liquid nitrogen and stored at −80°C until purification. Prior to purification the cell lysates were thawed slowly on ice and the cells were lysed by sonication at 4°C. The lysates were cleared by centrifugation at 35,000 × g at 4°C for 60 min and filtered through a 0.45 μm filter.

bio-PP1c was purified by anion exchange chromotography on a 5 mL HiTrap Q HP column (GE Healthcare) equilibrated in 50 mM Tris pH 7.5, 100 mM NaCl, 0.1 mM EGTA, 1 mM MnCl_2_, 5 mM β-mercaptoethanol (buffer A) and eluted with a 1 M NaCl 20 CV gradient in buffer A. The proteins were analyzed on Bolt SDS-PAGE 4%–12% Bis-Tris gels (ThermorFisher Scientific) stained with InstantBlue (Expedeon) and the presence of a biotinylated PP1 was confirmed by a western blot using a Pierce High Sensitivity Streptavidin-HRP antibody (ThermoFisher Scientific (#21130); 1:40,000). The positive fractions were pooled and concentrated using a Vivaspin device with a 10 kDa cut-off membrane (Sartorius) to a volume suitable for gel filtration. HiLoad Superdex 200 PG 16/600 column (GE Healtcare) equilibrated in buffer A was used for the gel filtration and the positive fractions confirmed by SDS-PAGE and western blot, as before. This results in a partially pure protein; the relevant fractions were pooled and stored at −80°C in small aliquots. Full purification is reached in later stages due to the high affinity and specificity of the biotin to streptavidin (on the SPR chip).

His-PP1c was first purified by affinity chromatography on a 5 mL HisTrap HP column (GE Healthcare) pre-equilibrated with buffer A + 20 mM imidazole. After washing with 10 CV buffer A + 20 mM imidazole, proteins were eluted with 8 CV buffer A + 250 mM imidazole. The eluate was then onto a 5 mL HiTrap Q HP column (GE Healthcare), followed by size exclusion chromatography on a HiLoad Superdex 200 PG 16/600 (GE Healthcare), as before. The proteins were analyzed on Bolt SDS-PAGE 4%–12% Bis-Tris gels (ThermoFisher Scientific) stained with InstantBlue (Expedeon). Samples containing pure his-PP1c were pooled and concentrated, and stored at −80°C in small aliquots.

#### Binding of R15 to bio-PP1c

Partially purified bio-PP1c (100 μL) was incubated on neutravidin agarose beads (ThermoFisher Scientific) for 2 hr at 4°C with shaking in IP buffer (50 mM Tris pH 7.5, 100 mM NaCl, 0.1 mM EGTA, 0.05% Tween 20, 0.1% NP40). The beads were then washed three times with the IP buffer and incubated in the presence or absence of 10 μM R15 (A or B) over night at 4°C with shaking in IP buffer. The beads were then washed three times with IP buffer and bound proteins were eluted by boiling in 60 μL of Laemmli buffer. Bound proteins were then analyzed on Bolt SDS-PAGE 4%–12% Bis-Tris gels (ThermoFisher Scientific) stained with InstantBlue (Expedeon) and the presence of a biotinylated PP1c was confirmed by a western blot using a Pierce High Sensitivity Streptavidin-HRP antibody (ThermoFisher Scientific (#21130); 1:40,000).

#### Compounds stock solutions for *in vitro* studies

The acetate salt of all compounds used in assays was dissolved in 100% DMSO and kept as a 50 mM stock (unless otherwise specified). Stock solutions were aliquoted and stored at −20°C.

#### Surface Plasmon Resonance (SPR)

##### Capture of bio-GBZ or bio–PP1c on the SA Sensor Chip

A Biacore T200 (GE Healthcare) system was used for all experiments and biotinylated GBZ (bio-GBZ) (Tsaytler et al., 2011) or bio-PP1c was captured on a Sensor Chip SA (GE Healthcare). The streptavidin coated surface was activated by 1 min injection with a solution of 50 mM NaOH and 1 M NaCl three times at a flow rate of 10 μL/min. bio-GBZ or bio–PP1c was diluted in the running buffer (50 mM Tris pH 7.5, 100 mM NaCl, 0.1 mM EGTA, 0.05% Tween 20, 0.1% DMSO) and injected at approximately 300 nM concentration at a flow rate of 10 μL/min directly to streptavidin coated surface to reach immobilization level of bio-GBZ or bio–PP1c corresponding to ∼200 and :6,000 RU, respectively. A blank immobilization was performed for one of the SA sensor chip surface to use as a reference.

##### Determining steady-state binding constants of R15 to bio-GBZ

With minor deviations, the same procedure and conditions were used in all binding experiments. Prior to determining binding constants, serial dilutions of 12 concentrations of the proteins were prepared in the running buffer in a 96-well plate. The protein dilutions were injected onto the surface of the chip at a flow rate of 30 μL/min, contact time was 1 min and dissociation time 2 min. After each injection, the surface was regenerated using 50 mM NaOH for 30 s. Maltose Binding Protein (MBP) was used as a control and showed low affinity for bio-GBZ or possibly non-specific binding (Figure S2A).

##### Determining steady-state binding constants of small molecules to R15 holophosphatase complexes using the bio-PP1c surface

With minor deviations, the same procedure and conditions were used in all binding experiments. Small molecules were stored as 50 mM stock solutions in 100% DMSO. Prior to determining binding constants, serial 1:1 dilutions, starting at 25 μM, of the compounds were prepared in the running buffer in a 96-well plate. If necessary the concentration range was adjusted to accurately determine the K_D_ depending on the initial results. Prior to each compound dilution series, the regulatory subunit, MBP-R15A^325-636^-His or MBP-R15B^340-698^-His, was diluted to 10 μM in the running buffer and captured on the bio-PP1c surface at a flow rate of 30 μL/min for 1 min to form the holophosphatase complex on the sensor chip surface. This was followed by 1 min stabilization period, to wash off any unspecific binding. Then, without regenerating the surface, the compound dilution series was injected onto the surface of the chip at a flow rate of 30 μL/min for 1 min, followed by 2 min dissociation time. After each dilution series, the surface was regenerated using 3 M NaCl for 90 s. After regeneration, SPR responses generally returned close to base levels and the bio-PP1c surface was ready for another capture and the next compound dilution series. Buffer blanks were also included for each dilution series for double referencing (Myszka, 1999). In order to be able to correct for small variations in DMSO concentration between samples, eight solvent samples ranging from 0.06 to 8% DMSO were injected every 50th cycle. The flow cell temperature was 20°C.

##### Data analysis

Sensorgrams were analyzed using the Biacore T200 evaluation software and the binding constants determined based on a steady-state model. Solvent correction was first applied where applicable to the samples to correct for the effects of DMSO (Frostell-Karlsson et al., 2000) and the reference flow cell subtracted. The buffer blanks were also subtracted from the sample data, this is referred to as double referencing and removes systematic artifacts, such as drift and contributions from systematic injections (Myszka, 1999). Kinetic experiments are carried out using different concentrations of analyte and their respective equilibrium binding levels determined. These equilibrium response levels (R_eq_) are plotted against concentration and fitted using a global fit, which is able to determine steady-state affinity constants, i.e., the concentration at 50% saturation is K_D_ (Frostell-Karlsson et al., 2000).

The SPR signal is a combination of many parameters, for example target coupling density, target molecular weight and analyte molecular weight (Giannetti, 2011). For comparing the steady-state affinity curves of R15 to bio-GBZ or the compounds to the holophosphatases, the data was normalized based on the R_max_ (maximum binding capacity of the surface based on the respective steady-state curve). If the steady-state curve had not reached saturation, then the extrapolated R_max_ from the Biacore evaluation software was used. Representative results of at least three independent experiments are shown. Data are means ± SD, n = 3 (except n = 5 for R15A binding to bio-GBZ and n = 4 for GBZ binding to R15A-PP1c).

##### Screening method

The binding of 69 derivatives of GBZ to R15-PP1c was measured using SPR. Derivatives of GBZ were designed through iterative chemistry cycles (to be reported elsewhere) by substitutions of the benzyl ring or changing the benzyl ring or by modifications to the aminoguanidine head or by substitutions in the bond between aminoguanidine and benzyl ring as well as substitutions of the aminoguanidine head. Two compounds were commercially available and 67 were custom synthesized.

During inspection of the sensorgrams, two compounds were excluded from the analyses due to compound aggregation on the surface of the SA chip. Four compounds could not be conclusively analyzed because of inconsistent results. Nine compounds showed no binding to R15-PP1c (Figure S4).

54 compounds bound to R15-PP1_C_ with affinities comprised between 0.03 to 46 μM. Most substitutions of GBZ resulted in a decreased affinity for R15A-PP1c and many had increased affinity for R15B-PP1c. For a compound to be deemed R15A or R15B selective, a filter of K_D_ at least 5x greater for one R15-PP1c holophosphatase over the other was set (Figure S4). Out of the 54 compounds with measurable binding to either R15A-PP1c or R15B-PP1c or both, eight compounds were selective for R15A-PP1c. Six of those compounds only bound R15A-PP1c and three of them, including GBZ, had a K_D_ < 10 μM. The other two selective R15A-PP1c compounds (including Sephin1) had a K_D_ < 5 μM for R15A and a K_D_ > 15 μM for R15B. Five compounds were selective for R15B-PP1c, among which four had a K_D_ < 1 μM but also bound to R15A-PP1c, albeit with a weaker affinity. Out of the five compounds selective for R15B-PP1c, Raphin1 was chosen for further studies due to its high affinity for R15B-PP1c and selectivity.

#### PP1c catalytic activity assay

Residual activity of PP1c was measured using the EnzChek Phosphatase Assay Kit (ThermoFisher Scientific) according to the supplier’s instructions, with minor modifications. Purified his-PP1c (30 nM) was incubated in 50 μL of 50 mM Tris pH 7, 1.5 mM EGTA, 3 mM MnCl_2_, 0.01% Brij-35, 0.15% β-mercaptoethanol with indicated final concentrations of CalyculinA (Cell Signaling), GBZ, Sephin1 and Raphin1 for 30 min at 4°C in a 96-well dark plate. 50 μL of the 200 μM DiFMUP working solution was added to each microplate well, mixed and incubated at room temperature (RT), protected from light, for 45 min. Fluorescence was measured using excitation at ∼360 nm and emission detection at ∼460 nm in a PHERAstar (BMG Labtech) microplate reader.

#### Protein analyses on Immunoblots

HeLa or MEF cells (90,000 cells/mL) were plated in 12-well plates (1 mL/well). The following day cells were treated as indicated; the medium was removed, fresh medium containing compound added and incubated at 37°C for the times indicated. The time course was performed in reverse order to facilitate collecting all samples at the same time. At the end of treatment cells were washed two times with ice-cold PBS and lysed in 150 μL Laemmli Buffer. Lysates were transferred to 1.5 mL eppendorf tubes, boiled at 95°C for 5 min, sonicated and resolved on Bolt SDS-PAGE 4%–12% Bis-Tris gels (ThermoFisher Scientific). Proteins were transferred to the nitrocellulose membrane using the iBlot 2 system (ThermoFisher Scientific). Membrane was blocked in 5% skimmed milk for 30 min and then probed with primary antibodies followed by incubation with the appropriate horseradish peroxidase-conjugated secondary antibodies (Promega). Proteins were visualized using ECL Prime (GE Healthcare) and imaged in the Chemi-Smart 5000 (Vilber-Lourmat, France) or ChemiDoc Touch (BioRad). Bands were quantified using ImageJ (NIH, USA). The following primary antibodies were used: e-IF2α-P (ThermoFisher Scientific (#44-728G), 1:1,000), e-IF2α (Abcam (#ab5369), 1:1,000), tubulin (Sigma-Aldrich (#T5168), 1:4,000), BiP (BD Biosciences PharMingen (#610978), 1:1,000), ATF4 (Santa Cruz Biotechnology (#sc-200), 1:500), Ppp1r15a (Proteintech (#10449-1-AP), 1:1,000), Ppp1r15b (Proteintech (#14634-1-AP), 1:1,000) and CHOP (ABR Affinity BioReagents (#MA1-250), 1:1,000).

#### Assessment of translation rates

HeLa or MEF cells (90,000 cells/mL) were plated in 12-well plates (1 mL/well). The following day cells were treated as indicated; the medium was removed, fresh medium containing compound added and incubated at 37°C for the times indicated. The time course was performed in reverse order to facilitate labeling all samples at the same time. After the treatment the cells were labeled with 100 μCi/mL ^35^S-methionine (Hartmann Analytic) for 10 min at 37°C (300 μL fresh medium containing 3 μL ^35^S-methionine was added to each well). Note that cells were not starved in methionine-free media before labeling with ^35^S-methionine for monitoring protein synthesis. The labeled cells were then washed twice with ice-cold PBS and lysed in 120 μL Laemmli Buffer. Lysates were transferred to 1.5 mL eppendorf tubes, boiled at 95°C for 5 min, sonicated and resolved on Bolt SDS-PAGE 4%–12% Bis-Tris gels (ThermoFisher Scientific). Gels were then stained with InstantBlue (Expedeon) and after the gels had been imaged they were transferred to a 20% ethanol, 7% acetic acid, 4% glycerol solution for 10 min. This was to prevent the gels from cracking while drying. The gels were transferred to filter paper and dried using a gel dryer. The gels were then exposed to a Storage Phosphor Screen (GE Healthcare) and analyzed by phosphorimaging using a Typhoon Imager Scanner (GE Healthcare).

#### Trypsin digestion

Trypsin digestions were carried out as previously described (Carrara et al., 2017). Purified R15s were diluted to 3 μM in PBS, and incubated for 15 min at RT with 100 μM compound, or DMSO vehicle. Reactions were initiated by addition of 5 nM of trypsin from bovine pancreas (Sigma-Aldrich), made up in PBS, and allowed to proceed for 5 min at 22°C, with shaking at 350 rpm. Digestion was stopped by addition of 4% Laemmli sample buffer and samples were run on 4%–12% NuPAGE Bis-Tris gels (ThermoFisher Scientific). Proteins were visualized by staining with InstantBlue Protein Stain (Expedeon).

#### eIF2α pull down experiments

Recombinant eIF2α was produced as described in Ito et al. (2004) and pull down experiments were carried out as previously described (Carrara et al., 2017). Amylose beads (New England BioLabs) were pre-equilibrated with interaction buffer (50 mM Tris pH 7.4, 200 mM NaCl, 0.05% Tween20). 20 μL amylose beads were incubated with purified MBP-tagged R15s (200 nM), P-eIF2α (1 μM) as appropriate, plus 200 μM Raphin1, or DMSO vehicle in a total volume of 200 μL. Input samples (5%) was removed at this step, and added to 4% SDS-Laemmli sample buffer. Beads were incubated for 10 min at 4°C with 20 rpm gentle rotation. The beads were washed 5 × 1 mL with interaction buffer, transferred to a fresh tube and finally resuspended with 50 μL 4% SDS-Laemmli sample buffer. Eluded beads were boiled and then run on 4%–12% NuPAGE Bis-Tris gels (ThermoFisher Scientific). Anti-MBP HRP (New England BioLabs (#E8038); 1:5,000) (to probe for R15s) and anti-eIF2α (AbCam (#ab26197); 1:1,000) antibodies were used for immunoblotting analyses of samples.

#### Selective holophosphatase activity assay

The assay was performed as previously described (Carrara et al., 2017). Dephosphorylation experiments were carried out in dephosphorylation buffer (50 mM Tris pH 7.4, 1.5 mM EGTA pH 8.0, 2 mM MnCl_2_), and all proteins were diluted to the appropriate concentration in dephosphorylation buffer. PP1c was diluted to 10 nM, R15s were diluted to 50 nM and P-eIF2α was diluted to 1 μM. Proteins were mixed as appropriate, and Raphin1 at 100 μM or DMSO vehicle control were added. Reactions were incubated at 30°C for 16 hr. 4% SDS-Laemmli sample buffer was added to stop the reactions. Samples were loaded on 4%–12% NuPAGE Bis-Tris gels (ThermoFisher Scientific), diluted 1-fold, and analyzed by immunoblotting using anti-Phospho-eIF2α (Ser51) (Cell Signaling (#9721); 1,000) and total anti-eIF2α (AbCam (#ab26197); 1:1,000) antibodies.

#### Monitoring Raphin1 stability

Raphin1, 10 μM (from a 10 mM DMSO stock), was added to 20 mL of pre-warmed water or cell culture media, in tightly-sealed 20 mL glass vials to reduce evaporation and incubated in an incubator at 37°C. 50 μL samples were taken at 0, 1, 2, 5, 7.5 and 10 hr and added to an Eppendorf tube containing 200 μL of chilled acetonitrile and internal standards. The samples were stored in a −20°C freezer until the end of the study. The samples were then analyzed by mass spectrometry using a TQ-S micro Triple Quadrupole from Waters and compared against a standard curve prepared in the same manner as the samples.

#### Monitoring cell viability

HeLa cells (40,000 cells/mL) were plated in 96-well plates (0.1 mL/well). The following day the cells were treated with different concentrations of Raphin1, in triplicate, as indicated and incubated at 37°C for 48 hr. To monitor cell death 1:2,000 dilution of the CellTox Green Dye (Promega) was added to the medium. The growth of the cells was monitored over time and pictures taken every 2 hr (two per well) with the IncuCyte ZOOM system (Essen BioScience). The default software parameters for a 96-well plate (Corning) with a 10x objective were used for imaging and the images were analyzed by the IncuCyte ZOOM software (Essen BioScience). The software was used to calculate the confluency from the two non-overlapping images of each well at each time point. The phase confluency refers to the percentage of the image area that is occupied by cells. The green confluency refers to the percentage of the image area that fluoresces green. The CellTox Green Dye (Promega) preferentially stains dead cells’ DNA, but is excluded from viable cells. To assess the effect of Raphin1 on cell viability, the percentage of dead cells was calculated as follows:

$$\%\;of\;\;dead\;cells=\frac{Green\;confluency\;\left(\%\right)\;at\;X\;hours}{Phase\;confluency\;\left(\%\right)\;at\;X\;hours}\times 100$$

#### Monitoring α2-adrenergic activity

α2-adrenergic activity was measured by Euroscreen. Adrenergic α2A (FAST-006A) receptor cells grown 18 hr prior to the test in media without antibiotics were detached by gentle flushing with PBS-EDTA (5 mM EDTA), recovered by centrifugation and re-suspended in “assay buffer” (DMEM/HAM’s F12 with HEPES + 0.1% BSA protease free). Cells were incubated at RT for at least 4 hr with Coelenterazine h (Molecular Probes). Dose response curves with the reference compounds were performed before testing the compounds. For agonist testing, 50 μL of cell suspension was mixed with 50 μL of test compound or reference agonist plated in a 96-well plate. The resulting emission of light was recorded using the Hamamatsu Functional Drug Screening System 6000 (FDSS 6000). Agonist activity of test compound was expressed as a percentage of the activity of the reference agonist at its EC100 concentration.

#### *In vivo* pharmacological treatments

The acetate salts of Raphin1 or GBZ were dissolved in water and sonicated for 10 min. The solution was aliquoted and kept at −20°C until use. Once thawed a tube was kept at 4°C and used within 24 hr. The compounds were administered by oral gavage.

Raphin1 was administered at 2 mg/kg (unless otherwise specified). Mice received a single dose or were treated chronically – one time per day. Control mice were treated with vehicle (water) only.

#### Collecting mice weight

Mice were weighted on a dedicated scale (A&D, FX3000iWP). 4 weeks old treatment and test naive C57BL/6J males and females (Figure 6B) and 2 months old treatment and test naive C3H/B6 males (Figure S6A) were used.

#### Videos of mice after Raphin1 administration

In order to assess the adrenergic side effects of treatment mice were monitored after a single administration of Raphin1. To this end 3 months old treatment and test naive C57BL/6J males were treated with Raphin1 (10 mg/kg) and left in a cage for 30 min. Spontaneous behavior of mice was recorded every 15 min. Mice similarly treated with GBZ (10 mg/kg) were used as a control.

#### Pharmacokinetics studies

Pharmacokinetics studies were performed by XenoGesis Ldt. Raphin1 was administrated orally in 6 weeks old treatment- and test-naive C57BL/6J males. Plasma samples were prepared by protein precipitation with methanol containing internal standards. Tissues were weighed and prepared by homogenization (1:3 in phosphate buffered saline) and protein precipitated with methanol containing internal standard. Following the addition of methanol, plasma and tissue samples were placed at −20°C for ≥ 1 hr (or overnight) to allow proteins to precipitate. The samples were then centrifuged at 2,500 x g (3,400 rpm) for 20 min at 4°C. The supernatants were analyzed by LC-MS/MS.

#### Glucose tolerance test

Treatment- and test-naive 4 weeks old C3H/B6 males were used. Raphin1 was administered to mice daily for 8 weeks before the test. After the overnight 14 hr starvation mice were injected intraperitoneally with D-glucose (Sigma-Aldrich) at 2 g glucose/kg body weight. Right before and at the indicated time points after the injection blood samples were collected from the tail vein and the blood glucose concentration was determined using The Accu-Chek Aviva Blood Glucose Meter (Roche).

#### Oil Red O staining

Treatment- and test-naive C57BL/6J males and females were used. Mice received a single dose of Raphin1 or cycloheximide (CHX, Sigma-Aldrich) at 40 mg/kg at 3 months of age or were treated chronically with Raphin1 at 2 mg/kg from 4 weeks of age for 10 weeks before the analysis. Mice were culled by cervical dislocation, liver was extracted, fresh-frozen in the O.C.T. compound (VWR International) and kept at −80°C until use. Oil Red O staining was performed on 10 μm thick frozen cryosections (Leica CM1850). Sections were washed in 60% propanol and stained for 20 min at room temperature with filtered 0.33% Oil Red O solution (VWR International). After rinsing with distilled water sections were counterstained with hematoxylin (VWR, diluted 1:15) for 1 min. Images were acquired with a 40X objective using SCN400F scanner (Leica).

#### Behavioral procedures

Morris water maze and the following fear conditioning were performed on C57BL/6J wild-type males aged 2.5 months. Mice were treatment- and test-naive before the treatment started. Treatment with Raphin1 started two weeks before and was continued during the behavioral tests.

##### Morris water maze

The water maze consisted of a white circular tank (1.50 m diameter) filled with opaque water adjusted to 21 ± 1°C. For the hidden platform task, the escape platform (10 cm diameter) was positioned 1 cm below water surface in the center of one of the pool quadrants. The walls surrounding the water maze were hung with posters and flags, which served as visual cues and were visible during all stages of 5 trainings and testing. Movement of the mice within the pool was tracked and analyzed with a computerized tracking system (ViewPoint, France). During training mice were required to locate a submerged hidden platform by using only extra-maze cues. Each mouse received five blocks of training trials over five consecutive days. In each trial mice were placed in the pool at one of four randomized start positions (NE, SE, SW, NW), and allowed to locate the hidden platform. Trials lasted for a maximum of 120 s and were separated by 15-20 min intervals. If a mouse failed to find the platform within this period, it was guided to its position. Spatial learning performance was assessed during a probe trial 1 hr after the last session of training, and for which the target platform was removed from the pool. To evaluate the performance during training trials the latency and distance traveled to find the hidden platform were measured. For the probe trial, the percentage of time in each quadrant was used as index of spatial learning performance.

##### Fear conditioning

Testing was performed in polymodal operant chambers (Coulbourn Instruments, Allentown, PA, USA). Each chamber (18.5 × 18 × 21.5 cm) consisted of aluminum side walls and Plexiglas rear and front (the door) walls. A loudspeaker and a bright light constituted the sources of the cues during conditioning and cue-testing. The general activity of animals was recorded through the infrared cell placed at the ceiling of the chambers and was directly recorded using the Graphic State software (Coulbourn). For conditioning, mice were allowed to acclimate for 4 min, then a light/tone (10 kHz) – Conditioned Stimulus (CS) was presented for 20 s and co-terminated by a mild (1 s, 0.4 mA) footshock – an aversive Unconditioned Stimulus (US). Mice were returned to their home cages 2 min later. Testing was performed 24 hr following conditioning session. Testing for the context was performed in the morning. The mouse was placed back into the same chamber that was used for the conditioning and allowed to explore it for 6 min without presentation of the light/auditory CS or US. Testing for the cue was performed in the afternoon (about 5 hr after the context testing). The contextual environment of the chambers was changed (wall color, odor and floor texture). Mouse was placed in the new chamber and allowed to habituate for 2 min and then presented with light/auditory cues for 2 min. This sequence was repeated. For both context and cue testing the percentage of time spent in the immobile position (% Freezing) was scored as an indicator of fear memory.

#### Quantitative RT-PCR

Quantitative RT-PCRs were performed on symptomatic 10 weeks old HD82Q males (cortex) and 6 months old CMT-1B males and females (sciatic nerve). RNA was extracted using RNeasy Mini Kit (QIAGEN) according to the manufacturer’s instructions. RNA concentration was measured using a NANODROP1000 spectrophotometer (ThermoFisher Scientific) and 0.5 μg RNA was reverse transcribed to cDNA using iScript cDNA Synthesis Kit (Bio-Rad). RNA level of indicated genes was assessed in the quantitative PCR on a Corbett Rotor-Gene version 6000 using SYBR® Select Master Mix (Applied Biosystems) and the following primers: *R15a* ((f) - GACCCCTCCAACTCTCCTTC, (r) – TCTCAGGTCCTCCTTCCTCA), *Chop* ((f) - GGAGAGAGTGTTCAAGAAGGAAGTG, (r) - GCAGGTCCTCATACCAGGCTT). RNA level is presented relatively to the house keeping gene *Gapdh* ((f) - TGGGTGGTCCAGGGTTTCTTACTCCTT, (r) – CGACTTCAACAGCAACTCCCACTCTTCC) and expressed as a fold change.

#### Treatment of HD^82Q^ mice with Raphin1

To produce an experimental group, HD^82Q^ transgenic males were crossed with C3H/B6 F1 wild-type females. 4 weeks old treatment- and test-naive transgenic mice and their wild-type littermate controls were randomized in different groups and treated with Raphin1 for the indicated time. Only males HD^82Q^ were used. Mice weight was collected weekly or bi-weekly.

#### Monitoring huntingtin level

Huntingtin levels were monitored in the HD^82Q^ mice subjected to the treatments indicated in the figures legends. Mice were culled by cervical dislocation, a part of cortex was extracted, fresh-frozen in liquid nitrogen and kept at −80°C until use. Tissue was homogenized and lysed in Laemmli Buffer (1 mL for 0.5 mg tissue). Lysates were boiled at 95°C for 5 min, sonicated and resolved on Bolt SDS-PAGE 4%–12% Bis-Tris gels (ThermoFisher Scientific). Proteins were transferred to the nitrocellulose membrane (Bio-Rad). Membrane was blocked in 5% skimmed milk for 30 min and then probed with primary antibodies followed by incubation with the appropriate horseradish peroxidase-conjugated secondary antibodies (Promega). Proteins were visualized using ECL Prime (GE Healthcare) and imaged in the Chemi-Smart 5000 (Vilber-Lourmat, France). SDS-resistant huntingtin assemblies were quantified using ImageJ (NIH, USA). The huntingtin assemblies were analyzed using the huntingtin 2B4 antibody (Euromedex (#HU-2B4), 1:1,000). The SDS-soluble huntingtin was revealed with the huntingtin antibody 1C2 (Euromedex (#PQ-1C2), 1:2,000).

#### Monitoring huntingtin inclusions

##### Immunofluorescence

Huntingtin inclusions were revealed in the HD^82Q^ mice subjected to the treatments indicated in the figures legends. Mice were culled by cervical dislocation, a brain was fresh frozen in the O.C.T. compound (VWR International) and sectioned (14 μm) using Cryostat (Leica CM3050 S). Tissue was fixed in 4% formaldehyde (ThermoFisher Scientific) and blocked in 5% normal goat serum (Millipore). Sections were stained overnight with the huntingtin antibody 2B4 (Euromedex (#HU-2B4); 1:1,000). Secondary antibody conjugated with Alexa 488 (ThermoFisher Scientific (#A11001); 1:400) was used for detection. The nucleus was counter stained with Hoechst 33342 (Lonza, 1:10,000).

##### Microscopy and quantification

For quantification, motor cortex was imaged using a Nikon TiE automated fluorescence microscope under the control of NIS Elements software (Nikon UK). Images were taken using a 40X 0.95 NA lens, fluorescence filter sets matched to Hoechst 33342 and Alexa 488 and an Andor Neo sCMOS camera. A regular grid of 3 by 4 fields covering a part of cortex was acquired. Each field was 346 microns by 410 microns. Images were analyzed using the ‘general analysis’ tool in NIS Elements. Inclusions were identified in the 2B4-labeled sections and nuclei were segmented in the Hoechst 33342 image. The total number of nuclei per field and the total number of inclusions inside nuclei per field were counted in each filed. An average of 383 nuclei per field was identified. The same threshold settings were used for all images. To check for systematic bias in our analysis due to variations in sample processing we also measured the average fluorescence intensity across the whole field. There we no measurable differences between samples observed.

In Figure 7E, sections were taken using Zeiss-710 confocal microscope. Images were obtained using a 63X 1.4 NA lens. The excitation laser wavelength, emission detection bands and pinhole diameter were chosen based on the manufacturers recommended settings for Hoechst 33342 and Alexa 488. The laser power and detector gain settings were adjusted to avoid saturation.

#### Monitoring translation rates *in vivo*

3 months old treatment- and test-naive C57BL/6J males were treated as indicated. 30 min before the end of treatment mice were injected intraperitoneally with 0.75 mCi/kg radioactive label – 75% ^35^S-methionine + 25% ^35^S-cystine (Hartmann Analytic). At the end of treatment mice were culled by cervical dislocation, brains were extracted, fresh-frozen in liquid nitrogen and kept at −80°C until use. Tissue was homogenized and lysed in Laemmli Buffer (1 mL for 0.5 mg tissue). Lysates were boiled at 95°C for 5 min, sonicated and resolved on Bolt SDS-PAGE 4%–12% Bis-Tris gels (ThermoFisher Scientific). Gels were then stained with InstantBlue (Expedeon) and after the gels had been imaged they were transferred to a 20% ethanol, 7% acetic acid, 4% glycerol solution for 10 min. This was to prevent the gels from cracking while drying. The gels were transferred to filter paper and dried using a gel dryer. The gels were then exposed to a Storage Phosphor Screen (GE Healthcare) and analyzed by phosphorimaging using a Typhoon Imager Scanner (GE Healthcare).

#### Compounds synthesis

The synthesis of compound C3 was previously reported (Carrara et al., 2017). The synthesis of Raphin1 is detailed below.

1. Synthetic procedure
  To a suspension of 2,3-Dichlorobenzaldehyde (22.00 g, 0.12570 mol) and aminoguanidine bicarbonate (17.1 g, 0.12570 mol) in Methanol (220 mL) was added acetic acid (22 mL) at 25°C. The resulting reaction mixture was heated at 70°C for the next ∼30 min. Upon heating the suspension became clear. Reaction completion was monitored on TLC using Dichloromethane/ Methanol (8/2) as mobile phase. After completion of reaction, the reaction mixture was allowed to cool down to 25°C and concentrated under vacuum. The resulting residue was suspended in diethyl ether (100 mL) and resulting product was collected by filtration. This process was repeated 3 times. At the end of the above process we could get the desired (E)-2-(2,3-dichlorobenzylidene)hydrazine-1-carboximidamide. LC-MS: m/z = 231.23 (M+H). The resulting product was also analyzed by 1H-NMR, 13C-NMR, potentiometric titration, HPLC and CHN analysis.
2. Proton NMR (1H NMR)
3. Proton NMR-D2O exchange
4. LCMS analysis

### Quantification and Statistical Analysis

Sample size for each experiment was estimated based on previous studies. All data of cellular and animal studies are shown as mean ± SEM, otherwise data from *in vitro* studies are shown as mean ± SD. For SPR graphs, individual data points are plotted. In the animal studies n represents sample size (the number of mice), whereas in the *in vitro* studies n represents biological replicates. The comparisons were carried out in GraphPad Prism 7 using unpaired two-tailed Student t test or two-way ANOVA with Tukey’s multiple comparisons test. Differences were considered statistically significant at p values below 0.05. Statistical methods used and significant differences are indicated in the corresponding figures. No samples, mice or data points were excluded from the analyses, except from one mouse excluded from the vehicle treated group in the Morris water maze due to thigmotaxis. Mouse experiments were performed in a blinded fashion.

## Supplemental Information

Method S1. Proton NMR Spectroscopy of Raphin1

Method S2. Spectroscopic Characterization of Raphin1 by Proton NMR-D2 Exchange

Method S3. LCMS Analysis of Raphin1
