# Supplementary material for: Target-Based Discovery of an Inhibitor of the Regulatory Phosphatase PPP1R15B
Source: Cell. 2018 Aug 23;174(5):1216–1228.e19. doi: 10.1016/j.cell.2018.06.030 (PMC6108835; doi:10.1016/j.cell.2018.06.030)
Supplement: Method S1. Proton NMR Spectroscopy of Raphin1 [file mmc4.pdf]

AAA_proton DMSO {Z:\2014\JUN 14} O2B 2

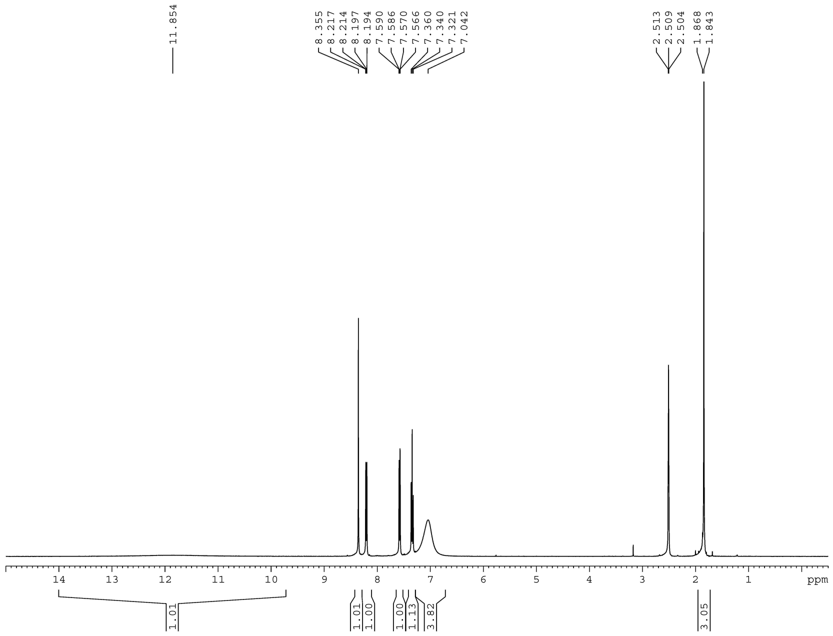


```
Current Data Parameters
NAME      27626-MANTIS-9
EXPNO                  1
PROCNO                 1

F2 - Acquisition Parameters
Date_           20140630
Time               15.15
INSTRUM            spect
PROBHD   5 mm BBO BB/19
PULPROG             zg30
TD                 39998
SOLVENT             DMSO
NS                    24
DS                     0
SWH            10000.000 Hz
FIDRES          0.250012 Hz
AQ             1.9999501 sec
RG                193.66
DW                50.000 usec
DE                  6.50 usec
TE                 293.9 K
D1            1.00000000 sec
TD0                    1

======== CHANNEL f1 ========
NUC1                  1H
P1                 14.00 usec
PLW1         10.50000000 W
SFO1         400.1324710 MHz

F2 - Processing parameters
SI                 65536
SF           400.1300000 MHz
WDW                   EM
SSB      0
LB                  0.30 Hz
GB       0
PC                  1.00
```
