# Supplementary material for: Target-Based Discovery of an Inhibitor of the Regulatory Phosphatase PPP1R15B
Source: Cell. 2018 Aug 23;174(5):1216–1228.e19. doi: 10.1016/j.cell.2018.06.030 (PMC6108835; doi:10.1016/j.cell.2018.06.030)
Supplement: Method S2. Spectroscopic Characterization of Raphin1 by Proton NMR-D2 Exchange [file mmc5.pdf]

D2O EXCHANGE
AAA_proton DMSO {Z:\2014\JUN 14} O2B 2

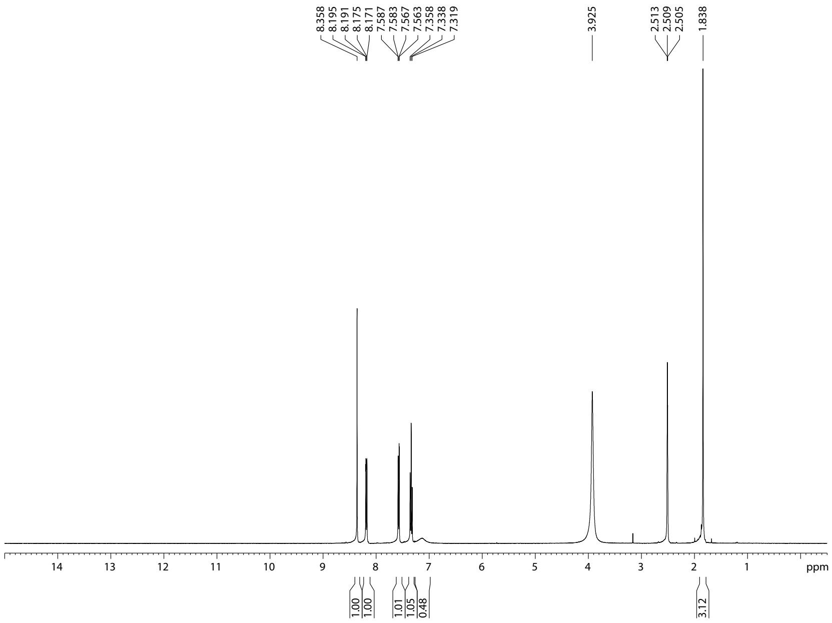

Current Data Parameters
NAME 27626–MANTIS–9
EXPNO 2
PROCNO 1

F2 – Acquisition Parameters
Date_ 20140630
Time 15.18
INSTRUM spect
PROBHD 5 mm BBO BB/19
PULPROG zg30
TD 39998
SOLVENT DMSO
NS 24
DS 0
SWH 10000.000 Hz
FIDRES 0.250012 Hz
AQ 1.9999501 sec
RG 193.66
DW 50.000 usec
DE 6.50 usec
TE 294.0 K
D1 1.00000000 sec
TD0 1

======== CHANNEL f1 ========
NUC1 1H
P1 14.00 usec
PLW1 10.50000000 W
SFO1 400.1324710 MHz

F2 – Processing parameters
SI 65536
SF 400.1300000 MHz
WDW EM
SSB 0
LB 0.30 Hz
GB 0
PC 1.00
