# Supplementary material for: Target-Based Discovery of an Inhibitor of the Regulatory Phosphatase PPP1R15B
Source: Cell. 2018 Aug 23;174(5):1216–1228.e19. doi: 10.1016/j.cell.2018.06.030 (PMC6108835; doi:10.1016/j.cell.2018.06.030)
Supplement: Method S3. LCMS Analysis of Raphin1 [file mmc6.pdf]

LCMS Analysis Report

Sample Name:	MANTIS-9-O2B-719-C1-INT-1-78		
Sample Type:	Unknown	Acquired By:	LCMS-04
Vial:	2:A,2	Sample Set Name:	30062014_UCH52_ASD_DCP01
Injection #:	1	Acq. Method Set:	PDS_METHOD_C
Injection Volume:	0.40 ul	Processing Method:	PDS_METHOD_C,
Run Time:	4.0 Minutes	Channel Name:	226.0nm, 285.0nm, MS TIC
Project Name	JUNE30-06-2014	Proc. Chnl. Descr.:	SQ 1: MS Scan MS TIC,
Date Acquired:	6/30/2014 15:39:59 IST		
Date Processed:	6/30/2014 15:44:33 IST, 6/30/2014 15:44:40 IST, 6/30/2014 15:46:45 IST		

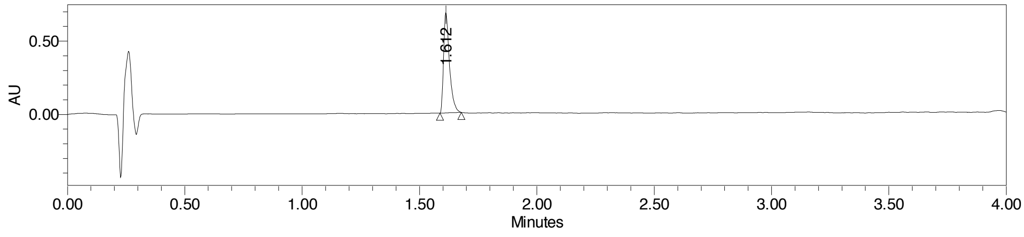

Channel Name 226.0nm; Channel PDA Spectrum

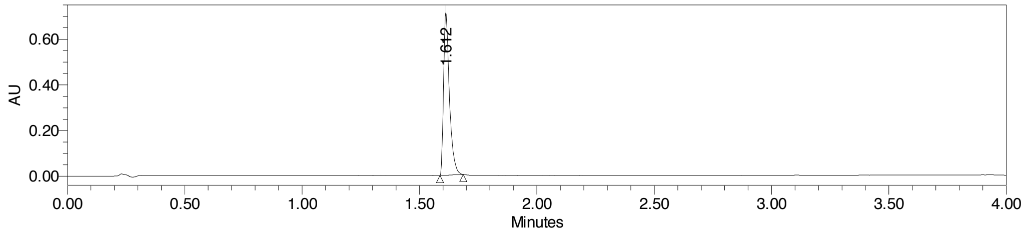

Channel Name 285.0nm; Channel PDA Spectrum

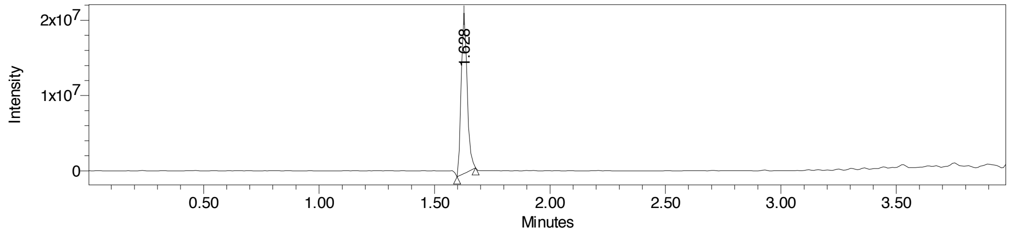

Channel Name MS TIC; Channel SQ 1: MS Scan

Peak Results
Channel: PDA Spectrum

	Retention Time (min)	Base Peak (m/z)	Height (µV)	Area (µV*sec)	% Area	Channel	Channel Name
1	1.612		709681	1231591	100.00	PDA Spectrum	285.0nm
2	1.612		683051	1188522	100.00	PDA Spectrum	226.0nm
